# An ncRNA transcriptomics-based approach to design siRNA molecules against SARS-CoV-2 double membrane vesicle formation and accessory genes

**DOI:** 10.1186/s12879-023-08870-0

**Published:** 2023-12-12

**Authors:** Rabia Nawaz, Muhammad Ali Arif, Zainab Ahmad, Ammara Ahad, Muhammad Shahid, Zohal Hassan, Ali Husnain, Ali Aslam, Muhammad Saad Raza, Uqba Mehmood, Muhammad Idrees

**Affiliations:** 1 Department of Biological Sciences, Superior University, Lahore, Pakistan; 2 Division of Molecular Virology, Center of Excellence in Molecular Biology, University of the Punjab, Lahore, Pakistan; 3 Vice chancellor, University of Peshawar, Peshawar, Pakistan

**Keywords:** SARS-CoV-2, siRNA, ncRNA, Non-structural genes, NSPs, Accessory genes, Open Reading frame ORF, Gene silencing

## Abstract

**Background:**

The corona virus SARS-CoV-2 is the causative agent of recent most global pandemic. Its genome encodes various proteins categorized as non-structural, accessory, and structural proteins. The non-structural proteins, NSP1–16, are located within the ORF1ab. The NSP3, 4, and 6 together are involved in formation of double membrane vesicle (DMV) in host Golgi apparatus. These vesicles provide anchorage to viral replicative complexes, thus assist replication inside the host cell. While the accessory genes coded by ORFs 3a, 3b, 6, 7a, 7b, 8a, 8b, 9b, 9c, and 10 contribute in cell entry, immunoevasion, and pathological progression.

**Methods:**

This in silico study is focused on designing sequence specific siRNA molecules as a tool for silencing the non-structural and accessory genes of the virus. The gene sequences of NSP3, 4, and 6 along with ORF3a, 6, 7a, 8, and 10 were retrieved for conservation, phylogenetic, and sequence logo analyses. siRNA candidates were predicted using siDirect 2.0 targeting these genes. The GC content, melting temperatures, and various validation scores were calculated. Secondary structures of the guide strands and siRNA-target duplexes were predicted. Finally, tertiary structures were predicted and subjected to structural validations.

**Results:**

This study revealed that NSP3, 4, and 6 and accessory genes ORF3a, 6, 7a, 8, and 10 have high levels of conservation across globally circulating SARS-CoV-2 strains. A total of 71 siRNA molecules were predicted against the selected genes. Following rigorous screening including binary validations and minimum free energies, final siRNAs with high therapeutic potential were identified, including 7, 2, and 1 against NSP3, NSP4, and NSP6, as well as 3, 1, 2, and 1 targeting ORF3a, ORF7a, ORF8, and ORF10, respectively.

**Conclusion:**

Our novel in silico pipeline integrates effective methods from previous studies to predict and validate siRNA molecules, having the potential to inhibit viral replication pathway in vitro. In total, this study identified 17 highly specific siRNA molecules targeting NSP3, 4, and 6 and accessory genes ORF3a, 7a, 8, and 10 of SARS-CoV-2, which might be used as an additional antiviral treatment option especially in the cases of life-threatening urgencies.

**Supplementary Information:**

The online version contains supplementary material available at 10.1186/s12879-023-08870-0.

## Background

An outbreak of a new strain of betacoronaviruses was detected in Wuhan city, which is the capital of Hubei province (China) in the month of December 2019. It was found to be responsible for respiratory tract infection [1, 2]. Clinical symptoms of the viral infection include fever accompanied by sore throat and respiratory distress [3]. This novel virus was later termed as Severe acute respiratory syndrome coronavirus-2 or simply SARS-CoV-2, in February 2020 [4]. According to World Health Organization report of April 2023, this potentially lethal virus has caused about 766,440,796 infection cases and 6,932,591 deaths across the globe [5]. SARS-CoV-2 is a highly mutable virus that has demonstrated a propensity to undergo genetic evolution. The emergence of novel mutations in its genomic sequence have been reported over time. These mutations arise in response to the virus adapting to new hosts, as it spreads within the populations [6].

SARS-CoV-2 is classified as an enveloped virus and is a member of the Coronaviridae family. Its genetic material approximately comprises of a 30 kilobase long, single-stranded positive sense RNA molecule [7]. It encodes for a total of 31 proteins, including 4 structural proteins, 11 accessory factors, and 16 non-structural proteins [8]. The structural makeup of SARS-CoV-2 is comprised of structural proteins including spike glycoproteins, envelope, membrane, and the nucleocapsid proteins. It also encodes for eleven accessory proteins, having key roles in the pathogenesis of virus [7, 8]. In addition, the non-structural proteins encoded by ORF1ab including NSP1-NSP16 also having an essential role in viral replication [9]. Upon entering the cell, SARS-CoV-2 takes control of host cell membranes organization and ultimately generates double membrane vesicles (DMVs) inside the Golgi apparatus accompanied by the aggregation of lipid droplets [10]. Similar to the mechanism followed by SARS-CoV [11–13], the double membrane vesicle formation of SARS-CoV-2 is facilitated by NSP3, in union with NSP4 and NSP6. The replication complexes of the virus are enclosed in these virus-induced organelles. Upon their release in cytoplasm, these replicative complexes facilitate the viral replication and proliferation [10]. On the other hand, accessory proteins of SARS-CoV-2 also play a critical role in viral entry inside the host cells, evasion of immune response and pathogenesis progression [14]. Accessory proteins are proven to have a role in interferon suppression also [14].

The biogenesis and assembly of double membrane vesicles along with viral replication mechanism are desired to be inhibited by silencing non-structural (NSP3, 4, and 6) and accessory genes (ORF3a, 6, 7a, 8, and 10) using an RNA interference technique. The RNA interference or RNAi mechanism, which involves post-transcriptional gene silencing or messenger RNA silencing, can be harnessed as an effective tool for down regulating the replicative pathways of viruses in human hosts [15]. RNAi employs short interference RNA and microRNA molecules for cleavage of specific sequences in the targeted viral mRNA. These short non-coding or ncRNA molecules, bind to their corresponding complementary sequences in mRNA molecules to inhibit their translation. Thus, RNAi mechanism ultimately results in silencing the expression of the viral genes [16, 17]. In silico approaches utilizing computational biology tools and databases have facilitated the design of siRNAs for targeted gene silencing [16–18]. Earlier in silico and in vitro studies reported siRNAs and miRNAs as an effective defense against many viruses including Hepatitis C virus [19], Human immunodeficiency virus [20], Influenza virus [21], Nipah virus [22], Zika virus [23], MERS-CoV [24, 25], and SARS-CoV-2 [16, 17, 26]. This in silico study includes conservation and phylogenetic analyses of NSP3, 4, and 6 sequences of SARS-CoV-2 strains across the globe along with the accessory genes including ORF3a, 6, 7a, 8, and 10. An ncRNA transcriptomics-based approach has been utilized for the designing of siRNA molecules against the selected non-structural as well as accessory genes of SARS-CoV-2 and further structural validations and verification of targeting specificity of the designed siRNA molecules have been assessed using different computational tools and algorithms.

## Methods

### Retrieval of gene sequences from NCBI

A total of one hundred SARS-CoV-2 strains across the globe were randomly selected from all the continents using NCBI Virus web portal^1^ and NCBI GenBank was used to obtain nucleotide sequences of NSP3, NSP4, and NSP6 from them (Additional file 1: Tables S1, S2). The SARS-CoV-2 isolate Wuhan-Hu-1 (accession number: NC_045512.2) was used as reference sequence. Subsequently, 17 whole-genome sequences of SARS-CoV-2 were obtained randomly and NCBI graphics was used to retrieve accessory gene sequences of ORF3a, ORF6, ORF7a, ORF8, and ORF 10. After sequence retrieval, NCBI ORF Finder^2^ [27] was used to screen the retrieved coding sequences (Additional file 1: Table S3). In order to check the similarity of accessory genes among other isolates of SARS-CoV-2, all CDS were individually subjected to NCBI BLASTn [28]. The nucleotide sequences of SARS-CoV-2 variants of concern were acquired for conservation analysis and checked against the designed siRNAs (Additional file 1: Table S2).

### Conservation and phylogenetic analysis across the globe

The sequences were aligned using MEGA11 [29] phylogenetic trees were constructed for each gene to predict the evolutionary divergence. Maximum likelihood method was used and bootstrap replications were kept as 1000. WebLogo application^3^ [30] was used to generate sequence logos for the selected gene sequences. BioEdit 7.2 and Jalview 2.11.2.0 programs were used in order to determine the consensus sequences.

### Target specific prediction of siRNAs

The obtained consensus sequences of selected genes were submitted to siDirect 2.0^4^ [31] for designing siRNAs. siDirect 2.0 is an online server that utilizes a fast and sensitive homology search algorithm to minimize any off-target effects and ensure functional siRNA design. Various parameters were set, including a melting temperature below 21.5 °C and GC content between 31.6 and 57.9%, along with the use of specific algorithms, such as Ui-Tei, Reynolds, and Amarzguioui combined rules to predict potential siRNAs for targeting the genes of interest. The stability of the seed-target duplex (T_m_) was also calculated to determine the RNA duplex’s formation ability.

### GC content calculations

To determine the exact GC content of predicted siRNAs accurately, a web based server known as ENDMEMO GC Content Calculator^5^ was employed.

### Validation of predicted siRNA molecules

To evaluate the efficacy and inhibiting potential of siRNA molecules, the siRNApred online server^6^ [32] was utilized. The 21-mer predicted siRNAs were subjected to screening against the Main21 dataset, using the support vector machine (SVM) algorithm and the binary pattern prediction approach. To further evaluate the predicted siRNA molecules, the i-Score Designer tool^7^ [33] was also employed using a second-generation algorithm for the calculation of i-scores and s-Biopredsi scores respectively.

### Heat capacity calculations

The siRNA-duplexes have a collective heat capacity (C_p_), and its melting temperature (T_m_C_p_) is determined as the local maximum of C_p_ curve when plotted against the temperature. For the determination of melting temperature at which the concentration of duplexes become half of their maximum value (referred to as T_m_(conc)), the DINAmelt Server^8^ [34] was used with the RNA option selected, including the option “Hybridization of two different strands”. The initial concentrations were set as 0.000005 M for siRNAs targeting NSPs and default for the accessory genes. All predicted siRNAs were analyzed using this method. The server generated the heat capacity values through the numerical differentiation of the ensembled free energy profiles, with respect to the temperatures.

### Prediction of secondary structures and minimum free energy calculations

The siRNA secondary structures were predicted using MaxExpect algorithm in RNAstructure program [35] as well as the respective free energy of folding. Default values were used for other parameters. Subsequently, RNA DuplexFold algorithm within the RNAstructure program was also utilized to calculate thermodynamics interaction between the viral siRNAs and their respective target sequences. The default values were maintained for other parameters including the maximum percent of energy difference and the maximum number of structures.

### Tertiary structure prediction and validation

The selected siRNAs, which passed the validations were further modelled using RNAComposer server^9^ [36] by their secondary structure in Vienna dot-bracket format. The model obtained was validated using the MolProbity server^10^ [37]. To find the most accurate 3D model, all-atom contacts and geometry, RNA backbone conformations, sugar puckers, Van der Waals forces, and H-bonds were analyzed. The tertiary structures of siRNA guide strands were viewed using UCSF Chimera (version 1.16) [38].

### Off-target minimization

Finally, to avoid any toxicity, the assessment of off-target binding effects of siRNA molecules, was made using NCBI nucleotide BLAST [28]. The siRNA sequences were screened against the Human Genomic + Transcript Database.

### Conservation analysis of designed siRNAs against SARS-CoV-2 variants

The target sequences of the designed siRNAs at each predicted position were aligned to gene sequences of SARS-CoV-2 variants of concern using MEGA11 [29] and were analyzed for conservation.

## Result

### Conservation and phylogenetic analysis across the globe

**Fig. 1:**
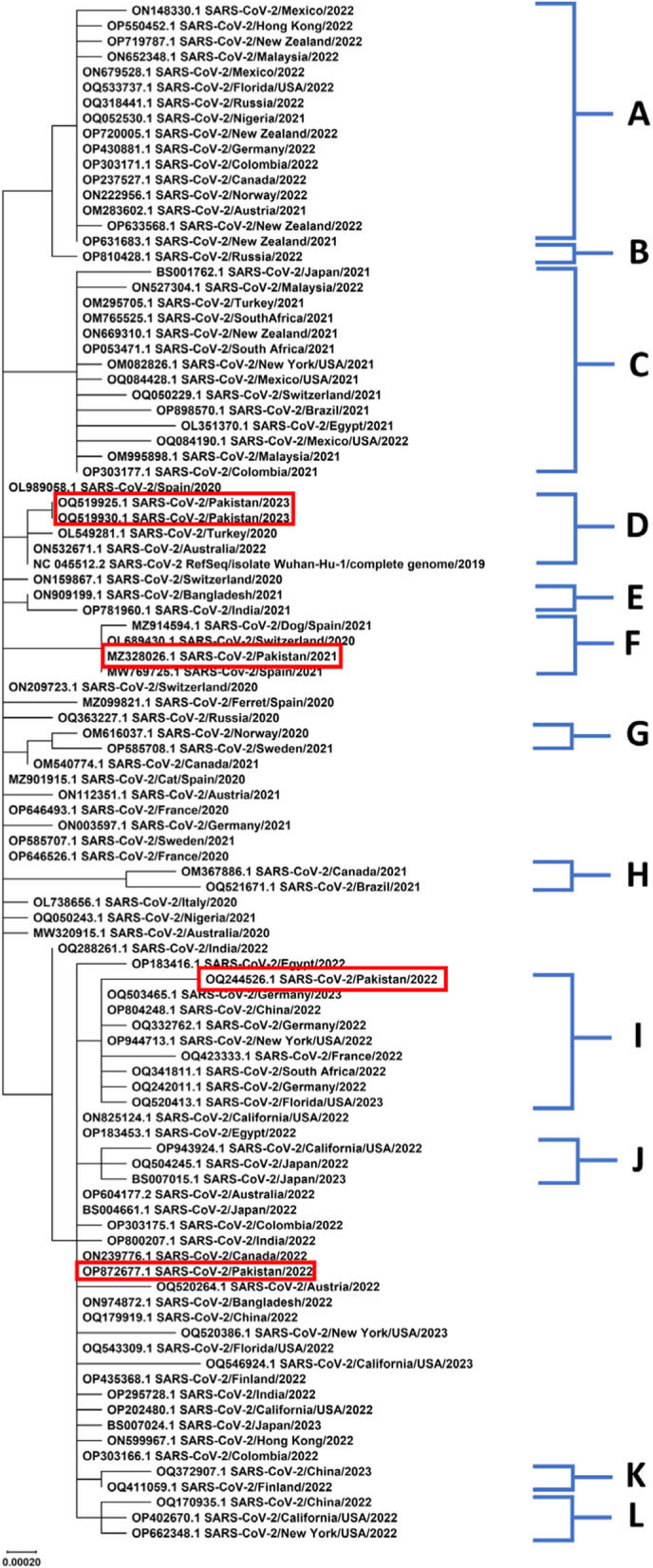
Phylogenetic analysis of NSP3 gene sequences of SARS-CoV-2

**Fig. 2:**
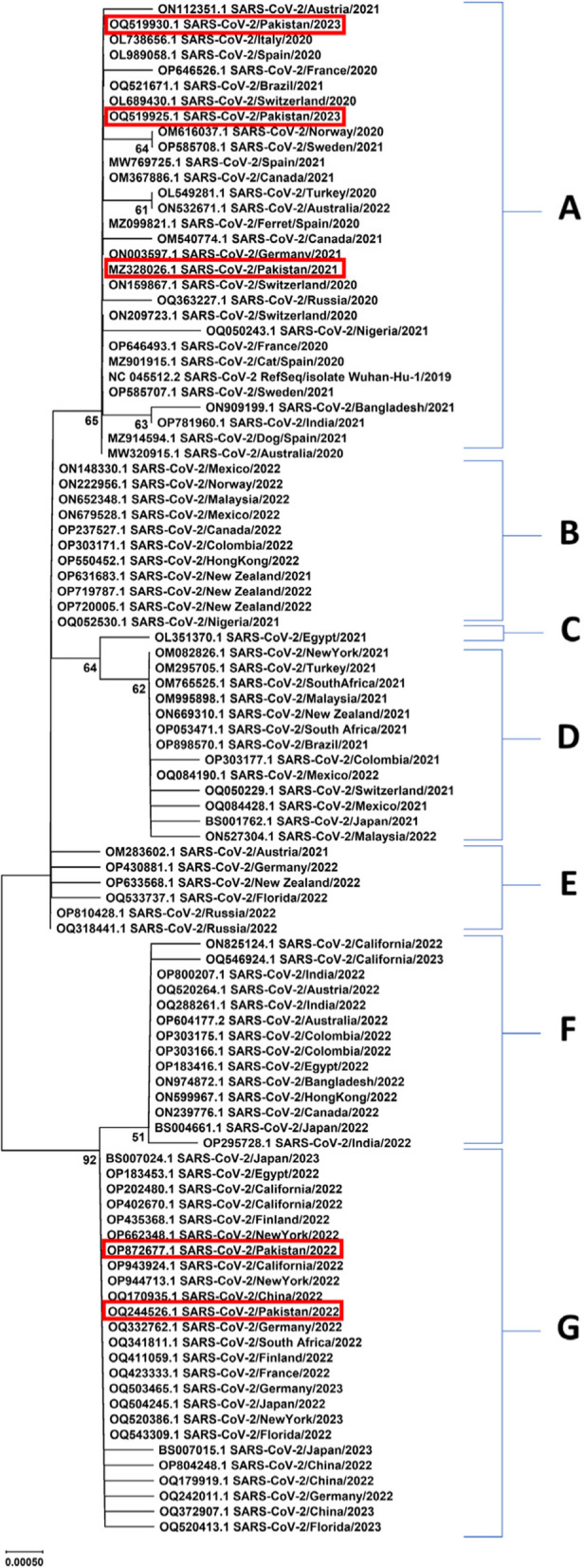
Phylogenetic analysis of NSP4 gene sequences of SARS-CoV-2

**Fig. 3:**
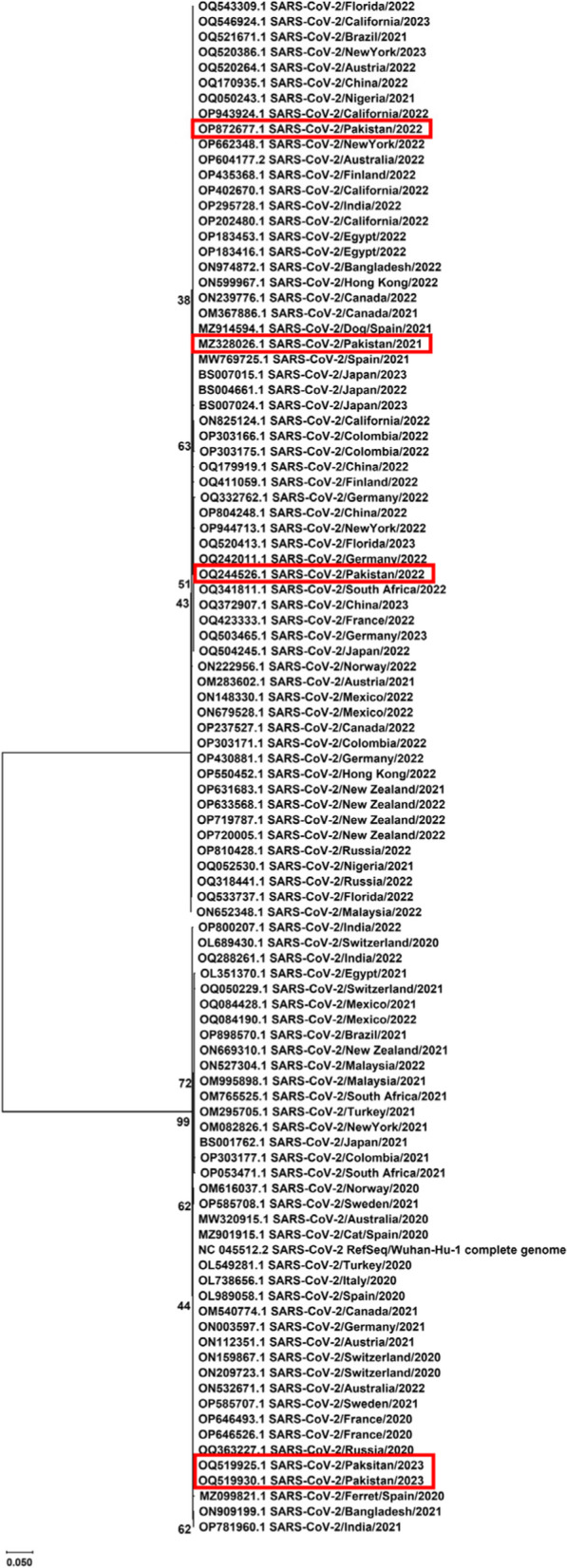
Phylogenetic analysis of NSP6 gene sequences of SARS-CoV-2

**Fig. 4:**
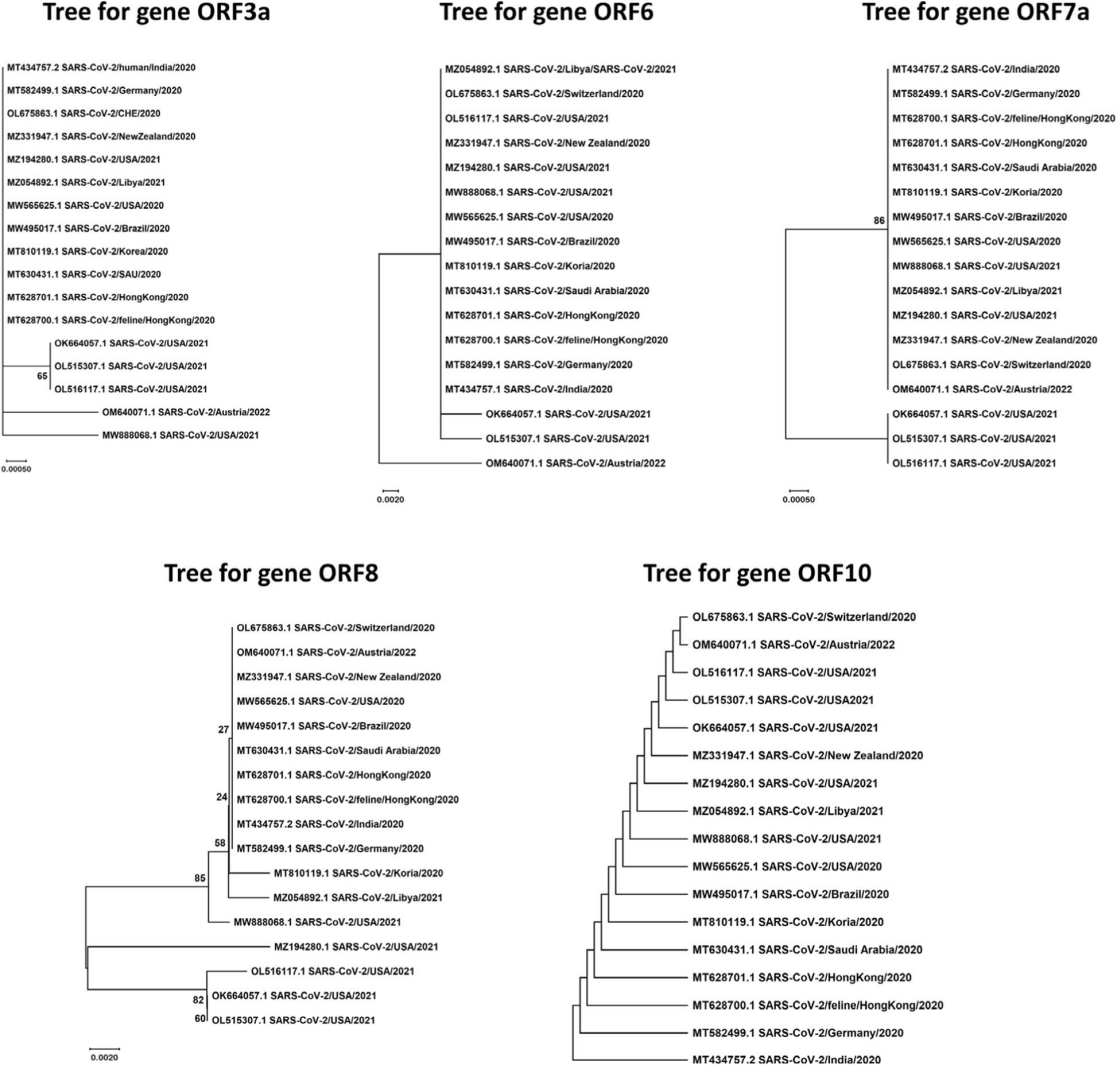
Phylogenetic analysis of accessory genes sequences of SARS-CoV-2

The multiple sequence alignments of non-structural and accessory gene sequences from different strains circulating in different countries revealed a high level of conservation. Phylogenetic trees of the selected gene sequences of NSPs and accessory genes were constructed using the Maximum Likelihood method and Tamura-Nei model (Figs. 1, 2, 3 and 4).

### Target specific prediction of siRNAs

The siDirect 2.0 web server utilized Ui-Tei, Amarzguioui, and the Reynolds algorithms to predict 41 siRNAs for the NSP3 gene, 12 siRNAs for the NSP4 gene, and 3 siRNAs for the NSP6 gene. For accessory genes, it predicted 7, 1, 2, 4, and 1 potential siRNA molecules for ORF3a, ORF6, ORF7a, ORF8, and ORF10 respectively. Notably, all the predicted siRNA molecules had a seed target duplex stability value (T_m_) below 21.5 °C, thus indicating potential minimization of off-target binding (Additional file 1: Table S4, S5, S6, S7).

### GC content calculations

The GC content of the predicted siRNA molecules ranged from 33.33 to 42.86% for the NSP3 gene, 33.33 to 45.24% for NSP4 gene, and 33.33 to 40.48% for the NSP6 gene (Additional file 1: Table S4, S5, S6). For accessory genes, the GC content ranged from 33.33–40.7, 35.71, 35.71–38.09, 38.09–42.85, and 35.71 for ORF3a, ORF6, ORF7a, ORF8, and ORF10 genes respectively (Additional file 1: Table S7).

### Validation based selection of efficient siRNA molecules

The effectiveness of predicted siRNA molecules was evaluated using siRNAPred.^11^ Overall, the siRNAs having binary scores equal or closest to 1 were selected as the most effective ones. A total of 12 out of 41 siRNA molecules predicted for NSP3, 2 out of 12 for NSP4, and 1 out of 3 for NSP6 met the criteria and were found to be highly effective. For accessory genes, the top 3, 1, 1, 3, and 1 potential siRNA molecules were found to be potential candidates for ORF3a, ORF6, ORF7a, ORF8, and ORF10 respectively. Primarily, these 24 siRNA molecules were selected for further analysis. The validity of predicted siRNAs was also confirmed using the i-Score Designer calculating the s-Biopredsi scores and i-Scores (Tables 1, 2).

**Table 1:** List of potential siRNA molecules targeting non-structural genes of SARS-CoV-2 and validation filters: The gene, target positions, target sequences, RNA oligo sequence of the finally selected siRNA molecules targeting NSP3, NSP4, and NSP6 genes of SARS-CoV-2 along with their GC content, minimum free energies of binding and folding, melting temperatures, binary validity scores, s-Biopredsi scores, and i-Scores

No.	Gene	Target position	Target sequence 21 nt target + 2 nt overhang	RNA oligo sequences 21 nt guide (5′ → 3′) 21 nt passenger (5′ → 3′)	GC content	∆MFE^**a**^	∆MFE^**b**^	T_**m**_C_**p**_	T_**m**_ (conc)	Binary score	s-Biopredsi Score	i-Score
					31.6–57.9*	< − 30*	> 1.5*	0–100*	0–100*	> 0.7*	< 1*	≥65*
					%	kcal /mol	kcal /mol	°C	°C			
**1**	NSP3	597–619	ACCAGTTGTTCAGACTATTGAAG	UCAAUAGUCUGAACAACUGGU CAGUUGUUCAGACUAUUGAAG	38.1	−34.8	1.5	81.2	80.0	1.008	0.830	66.9
**2**		682–704	GTGGAAGAAGCTAAAAAGGTAAA	UACCUUUUUAGCUUCUUCCAC GGAAGAAGCUAAAAAGGUAAA	35.71	−33.9	1.8	82.5	81.2	1.062	0.863	84.4
**3**		864–886	CGGACACAATCTTGCTAAACACT	UGUUUAGCAAGAUUGUGUCCG GACACAAUCUUGCUAAACACU	40.5	−35.0	1.5	81.1	79.9	1.072	0.807	67.3
**4**		1126–1148	AAGAGTGAAAAGCAAGTTGAACA	UUCAACUUGCUUUUCACUCUU GAGUGAAAAGCAAGUUGAACA	35.7	−31.4	1.8	82.3	80.8	1.030	0.874	78.6
**5**		1415–1437	TTGTTCAAGAGGGTGTTTTAACT	UUAAAACACCCUCUUGAACAA GUUCAAGAGGGUGUUUUAACU	35.7	−31.5	1.8	85.1	83.6	1.031	0.781	66.8
**6**		3917–3939	ATGGTTTAGCTGCTGTTAATAGT	UAUUAACAGCAGCUAAACCAU GGUUUAGCUGCUGUUAAUAGU	35.7	−32.5	1.8	84.1	82.8	1.064	0.837	70.9
**7**		5057–5079	TTGATAAAGCTGGTCAAAAGACT	UCUUUUGACCAGCUUUAUCAA GAUAAAGCUGGUCAAAAGACU	35.7	−31.9	1.7	82.7	81.6	1.064	0.814	67.5
**8**	NSP4	692–714	CTGGTGTTTGTGTATCTACTAGT	UAGUAGAUACACAAACACCAG GGUGUUUGUGUAUCUACUAGU	38.1	−34.2	1.8	82.2	80.9	1.001	0.873	78.8
**9**		713–735	GTGGTAGATGGGTACTTAACAAT	UGUUAAGUACCCAUCUACCAC GGUAGAUGGGUACUUAACAAU	40.5	−36.8	1.7	85.8	84.5	1.024	0.762	69.7
**10**	NSP6	372–394	AGCAAGAACTGTGTATGATGATG	UCAUCAUACACAGUUCUUGCU CAAGAACUGUGUAUGAUGAUG	38.1	−34.8	1.8	81.0	79.6	0.979	0.808	68.8

* Optimum filter values and ranges

^a^ Minimum free energy of siRNA-target hybridization

^b^ Minimum free energy of folding of siRNA guide strand

∆MFE Minimum free energy, T_m_C_p_ & T_m_ (conc) Melting temperatures

**Table 2:** List of potential siRNA molecules targeting accessory genes and validation filters: The gene, target positions, target sequences, RNA oligo sequence of the finally selected predicted siRNA molecules targeting ORF3a, ORF6, ORF7a, ORF8, and ORF10 genes of SARS-CoV-2 along with their GC content, minimum free energies of binding and folding, melting temperatures, binary validity scores, s-Biopredsi scores, and i-Scores

No.	Gene	Target position	Target sequence 21 nt target + 2 nt overhang	RNA oligo sequences 21 nt guide (5′ → 3′) 21 nt passenger (5′ → 3′)	GC content	∆MFE^**a**^	∆MFE^**b**^	T_**m**_C_**p**_	T_**m**_ (conc)	Binary score	s-Biopredsi Score	i-Score
					31.6–57.9*	< −30*	> 1.5*	< 100*	< 100*	> 0.7*	< 1*	≥65*
					%	kcal /mol	kcal /mol	°C	°C			
**1**	ORF3a	240–262	TTGCAACTTGCTGTTGTTGTTTG	AACAACAACAGCAAGUUGCAA GCAACUUGCUGUUGUUGUUUG	40.47	−32.9	1.5	84.5	83.4	0.980	0.840	69.7
**2**		550–572	TACCAGATTGGTGGTTATACTGA	AGUAUAACCACCAAUCUGGUA CCAGAUUGGUGGUUAUACUGA	40.47	−35.7	1.7	84.1	84.4	1.020	0.853	70.3
**3**		770–792	ATCCAGTAATGGAACCAATTTAT	AAAUUGGUUCCAUUACUGGAU CCAGUAAUGGAACCAAUUUAU	33.33	−32.2	1.9	81.6	83.4	0.992	0.869	78.0
**4**	ORF6	164–186	AGCAACCAATGGAGATTGATTAA	AAUCAAUCUCCAUUGGUUGCU CAACCAAUGGAGAUUGAUUAA	35.71	*−29.9*^*c*^	1.6	82.3	81.0	0.963	0.855	72.0
**5**	ORF7a	332–354	CACTTTGCTTCACACTCAAAAGA	UUUUGAGUGUGAAGCAAAGUG CUUUGCUUCACACUCAAAAGA	38.09	−33.2	1.5	86.2	84.6	0.921	0.830	75.9
**6**	ORF8	41–63	CTGCATTTCACCAAGAATGTAGT	UACAUUCUUGGUGAAAUGCAG GCAUUUCACCAAGAAUGUAGU	38.09	−34.0	*1.4*^*c*^	81.3	80.3	0.955	0.842	73.3
**7**		67–89	CAGTCATGTACTCAACATCAACC	UUGAUGUUGAGUACAUGACUG GUCAUGUACUCAACAUCAACC	40.47	−33.9	1.8	82.7	81.4	0.949	0.834	74.5
**8**		298–320	GTGCGTTGTTCGTTCTATGAAGA	UUCAUAGAACGAACAACGCAC GCGUUGUUCGUUCUAUGAAGA	42.85	−32.2	1.8	84.0	82.8	0.940	0.845	71.5
**9**	ORF10	20–42	TCGCTTTTCCGTTTACGATATAT	AUAUCGUAAACGGAAAAGCGA GCUUUUCCGUUUACGAUAUAU	35.71	−32.5	1.7	81.4	80.2	0.924	0.794	71.4

* Optimum filter values and ranges

a Minimum free energy of siRNA-target hybridization

b Minimum free energy of folding of siRNA guide strand

c Values not passing the validation criteria

∆MFE Minimum free energy, T_m_C_p_ & T_m_ (conc) Melting temperatures

### Heat capacity calculation

Melting temperatures T_m_(C_p_) and T_m_(Conc) for predicted siRNA molecules. The siRNA molecules exhibit greater effectiveness when their melting temperatures are elevated. For non-structural genes the T_m_(C_p_) values ranged from 81.0 to 85.8 °C and T_m_(Conc) values ranged from 79.6 to 84.5 °C (Table 1). For accessory genes, the T_m_(C_p_) values ranged from 81.3 to 86.2 °C and the T_m_(Conc) values ranged from 80.2 to 86.2 °C (Table 2).

### Secondary structure prediction and minimum free energy determination

**Fig. 5:**
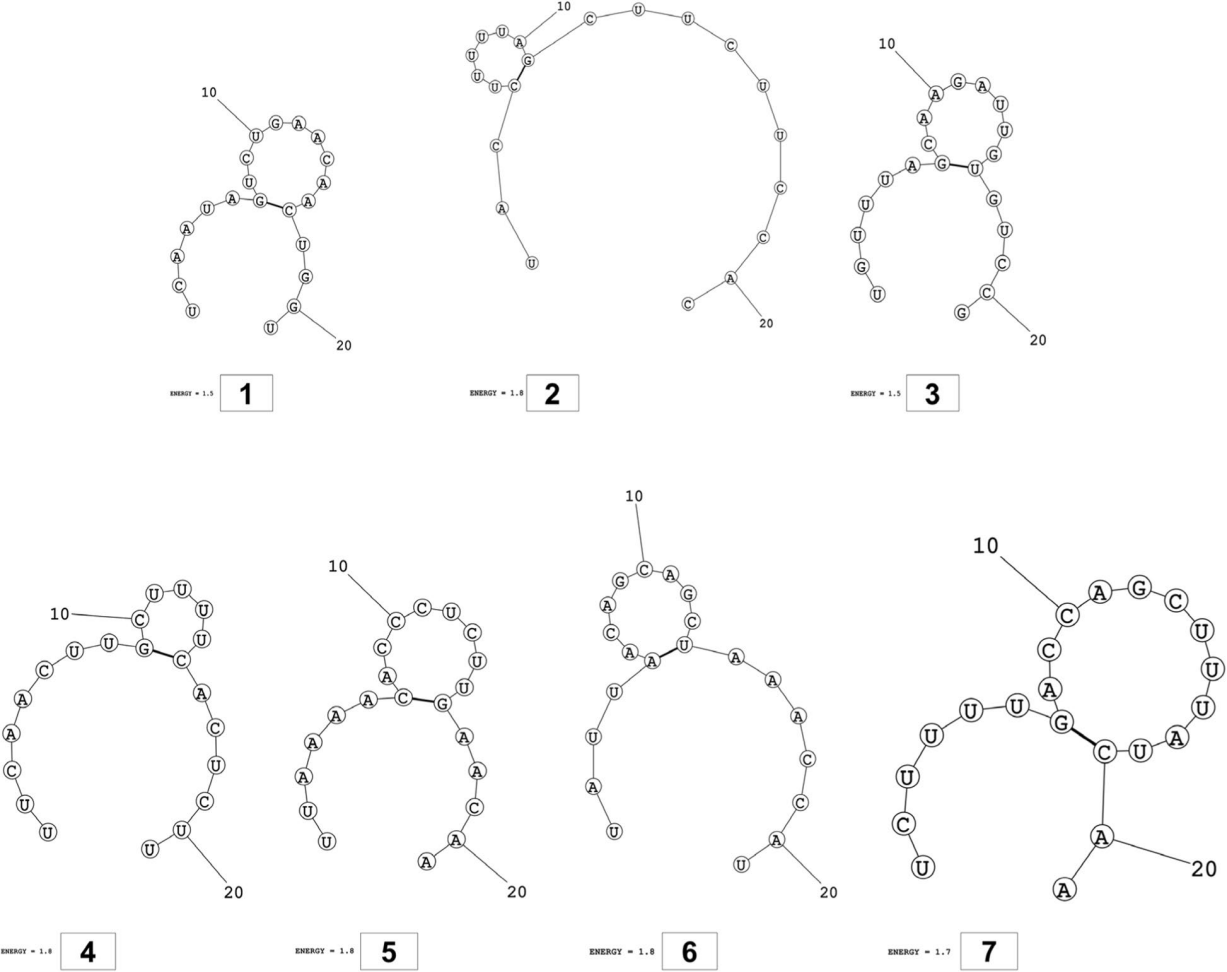
Secondary structures and MFE of siRNA molecules targeting NSP3

**Fig. 6:**
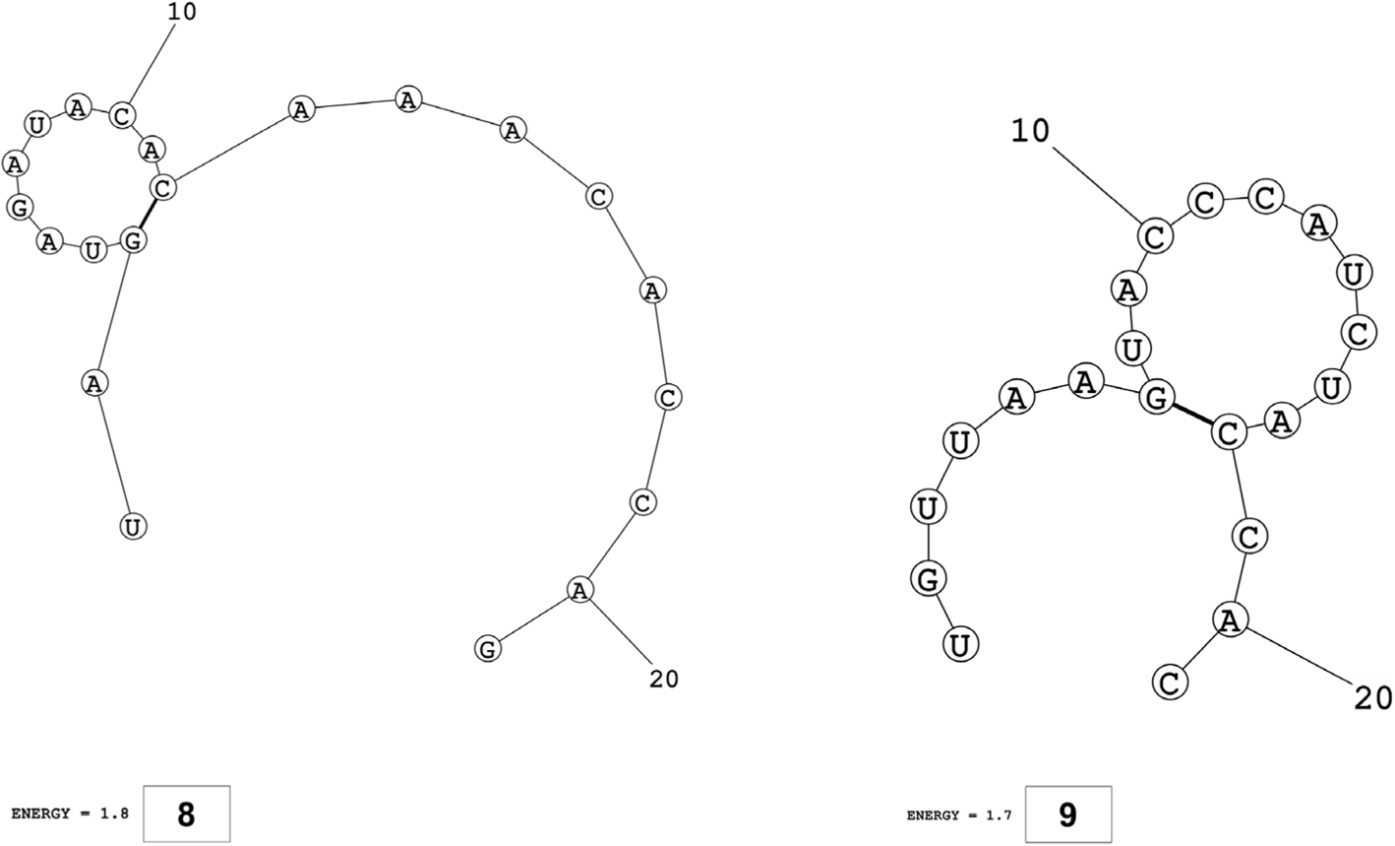
Secondary structures and MFE of siRNA molecules targeting NSP4

**Fig. 7:**
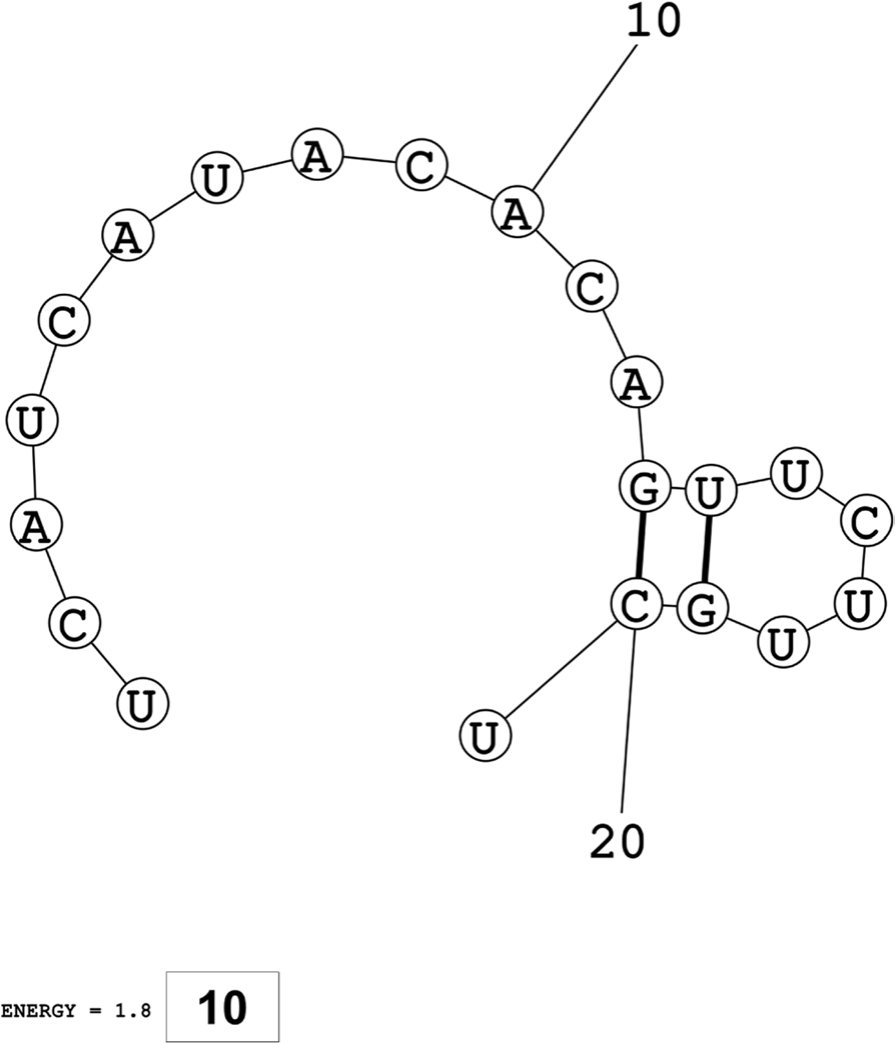
Secondary structure and MFE of siRNA molecule targeting NSP6

**Fig. 8:**
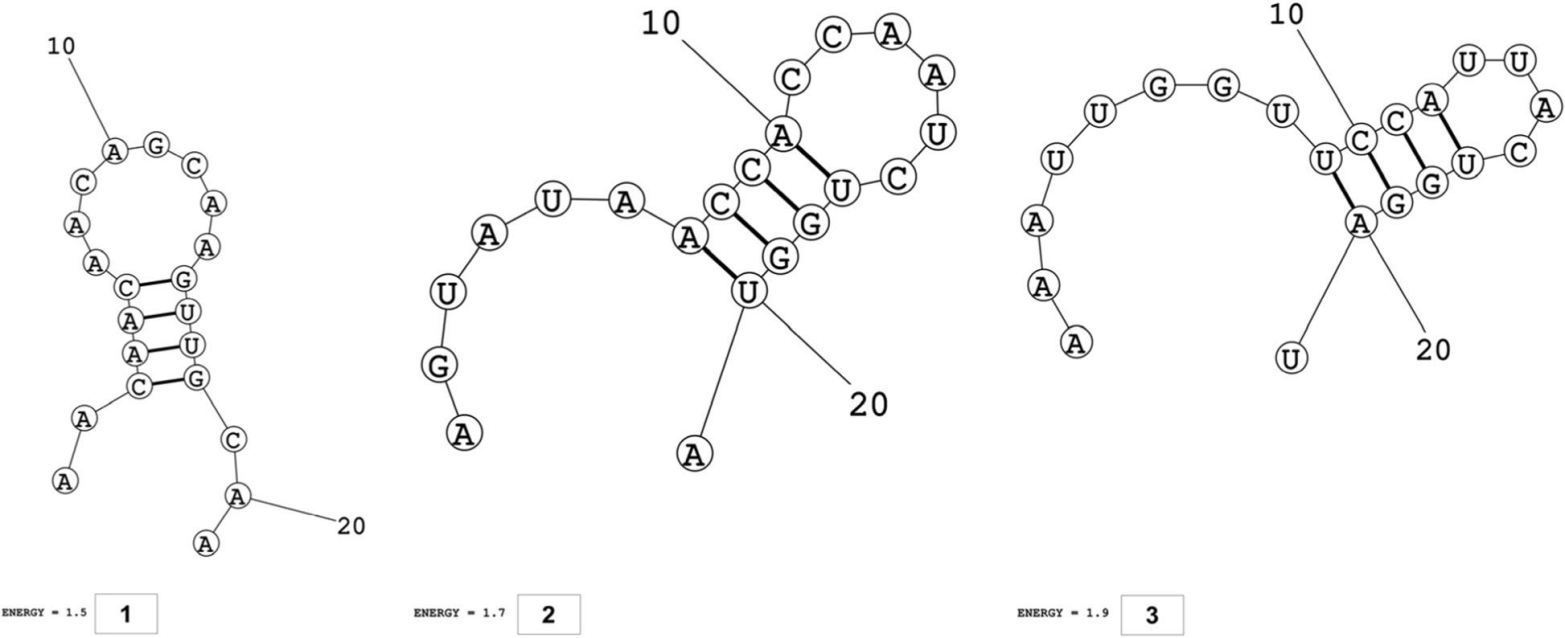
Secondary structures and MFE of siRNA molecules targeting ORF3a

**Fig. 9:**
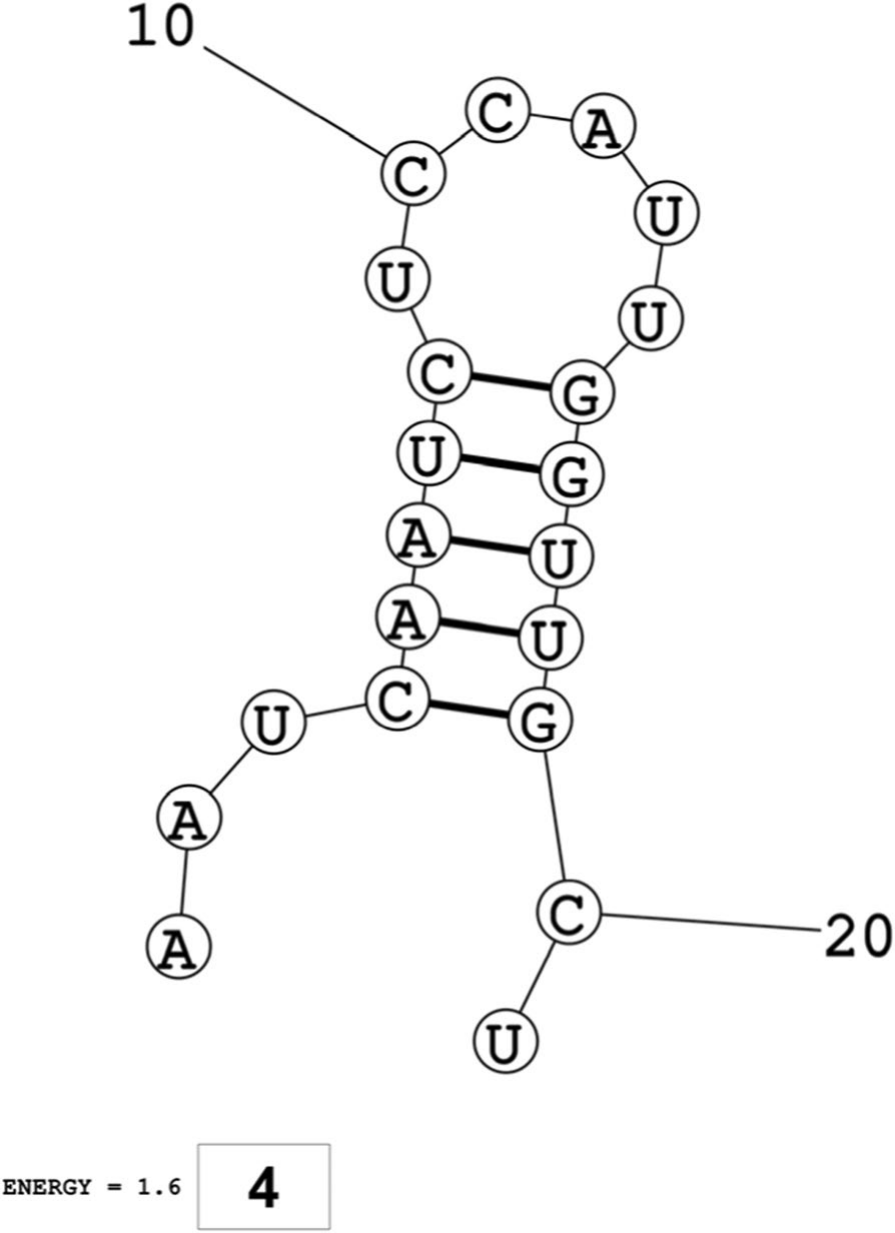
Secondary structure and MFE of siRNA molecule targeting ORF6

**Fig. 10:**
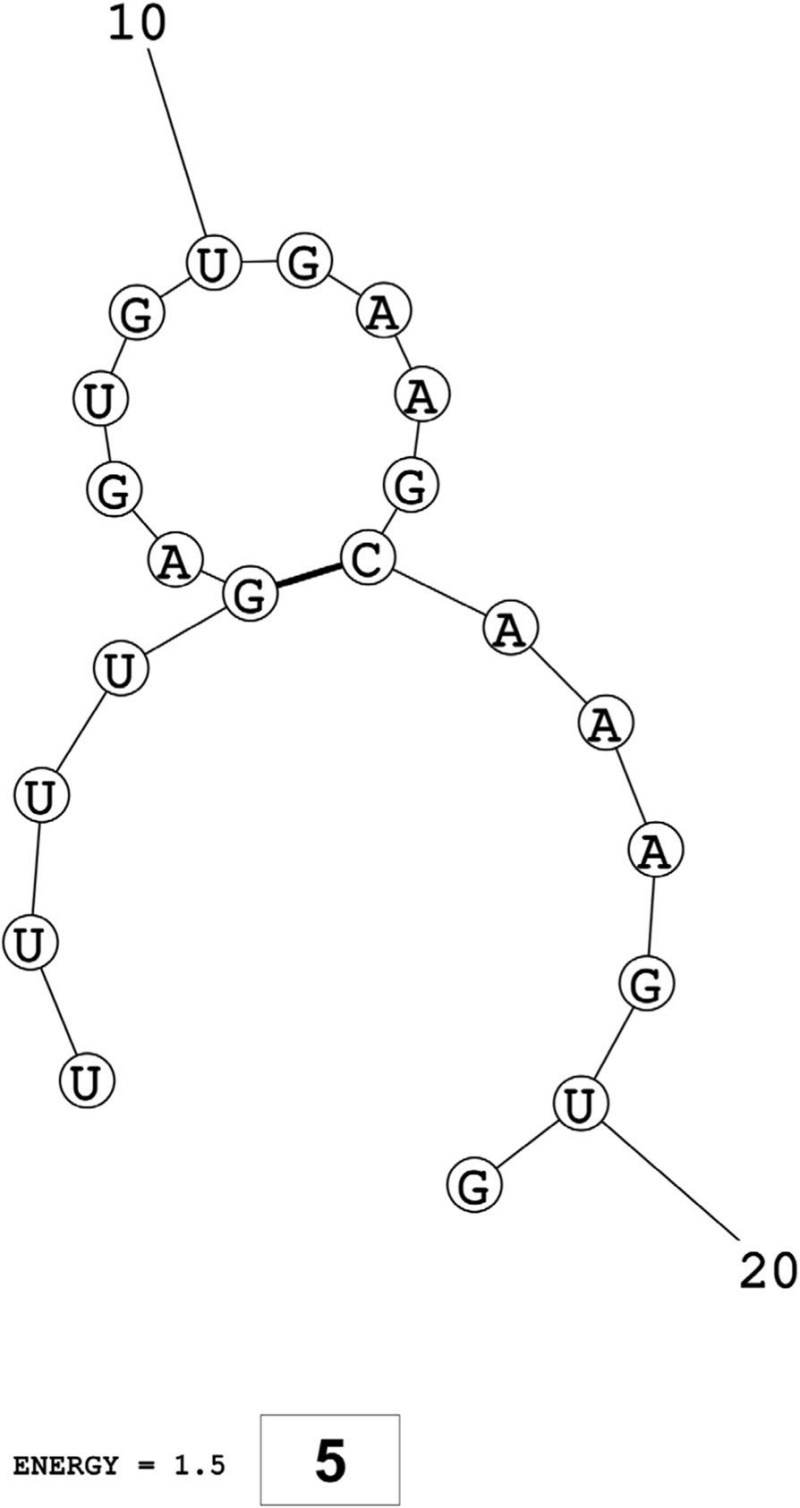
Secondary structure and MFE of siRNA molecule targeting ORF7a

**Fig. 11:**
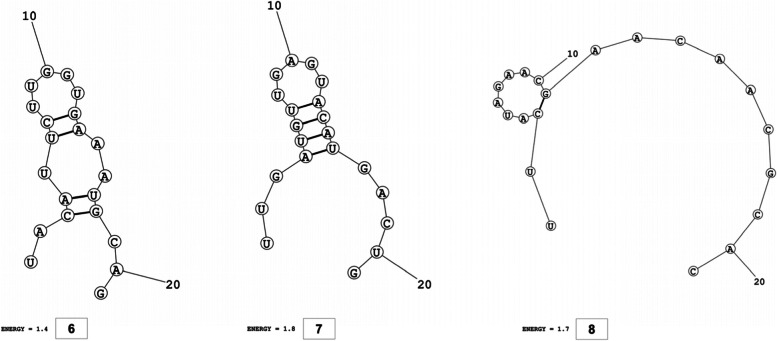
Secondary structures and MFE of siRNA molecules targeting ORF8

**Fig. 12:**
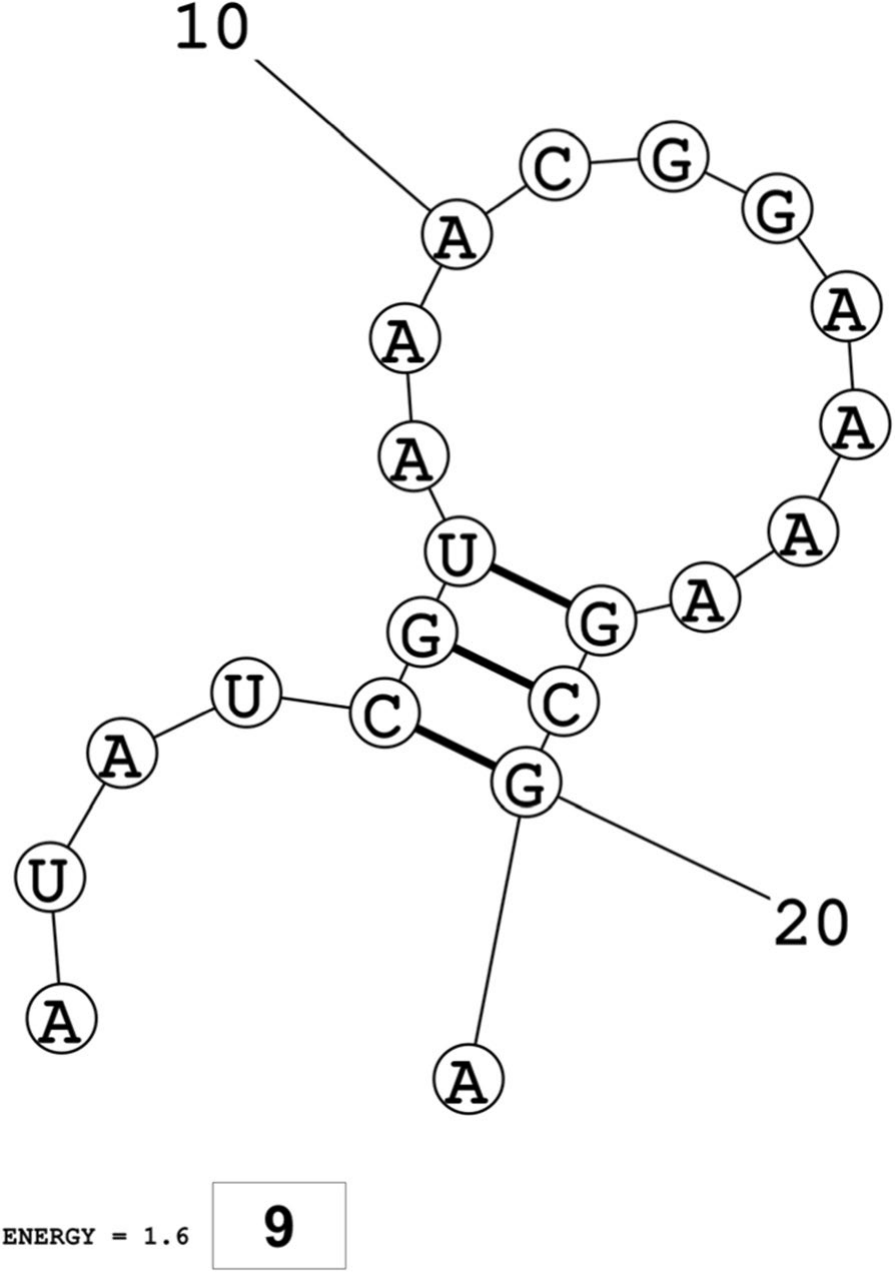
Secondary structure and MFE of siRNA molecule targeting ORF10

**Fig. 13:**
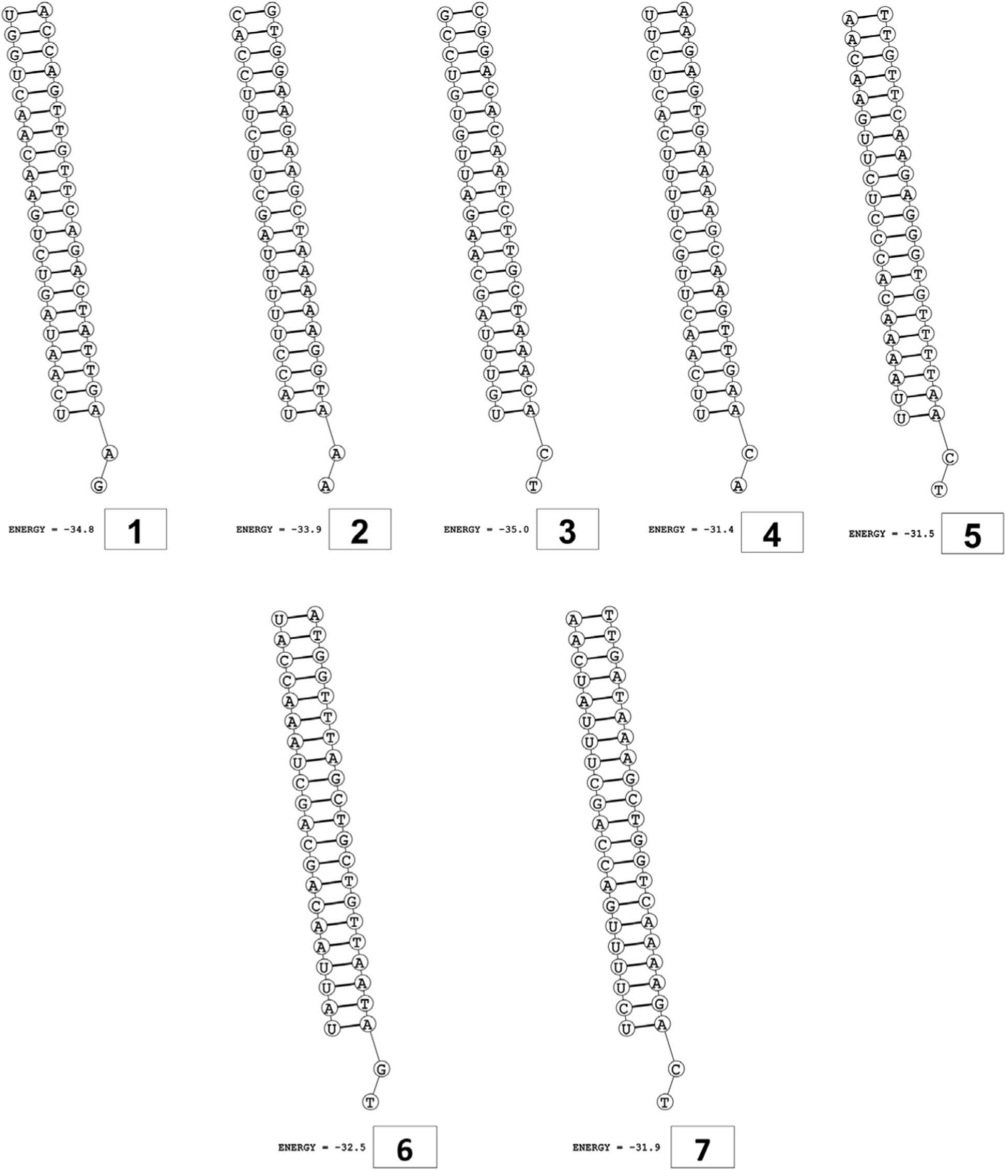
Secondary structures and MFE of siRNA-target duplexes for NSP3 regions

**Fig. 14:**
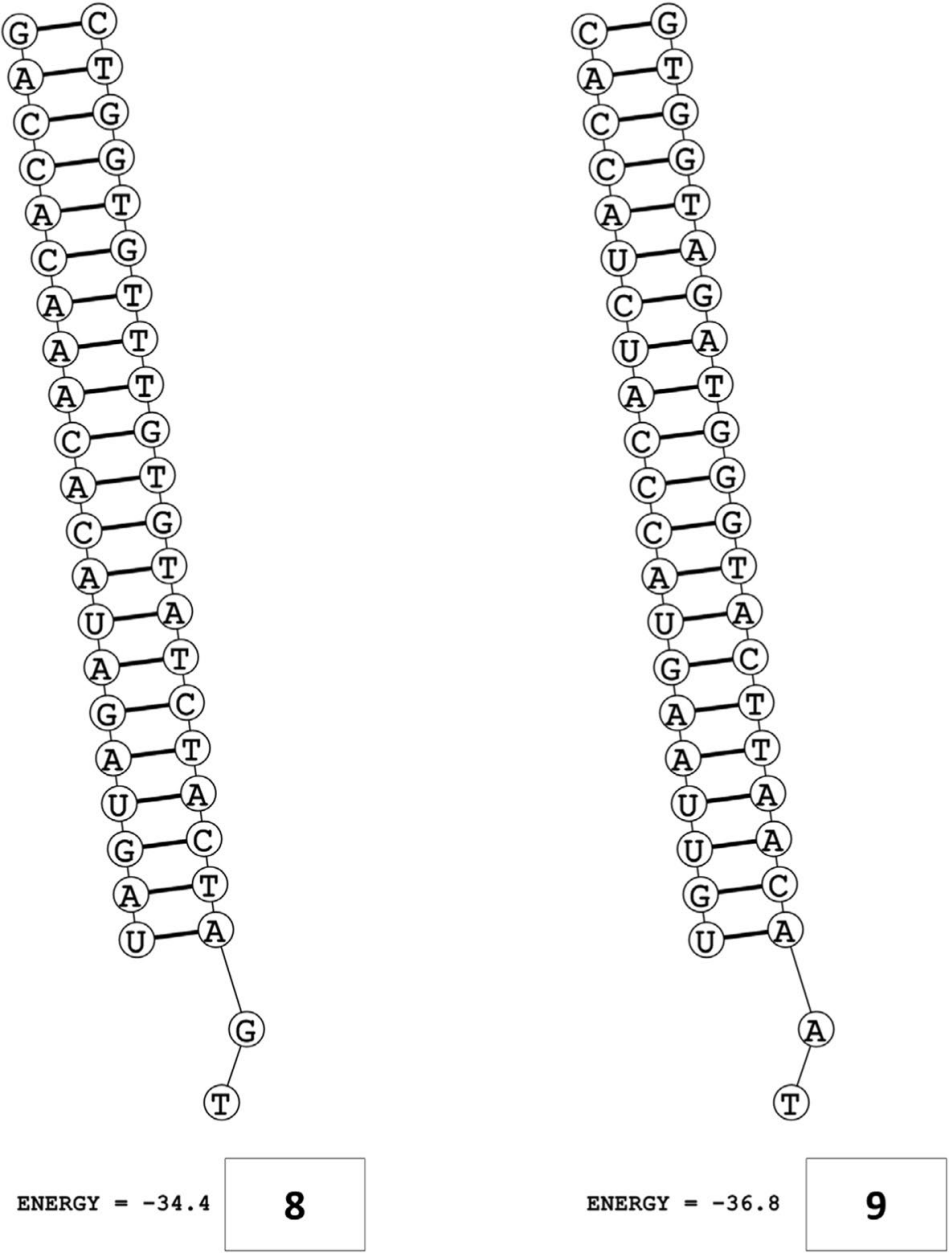
Secondary structures and MFE of siRNA-target duplexes for NSP4 regions

**Fig. 15:**
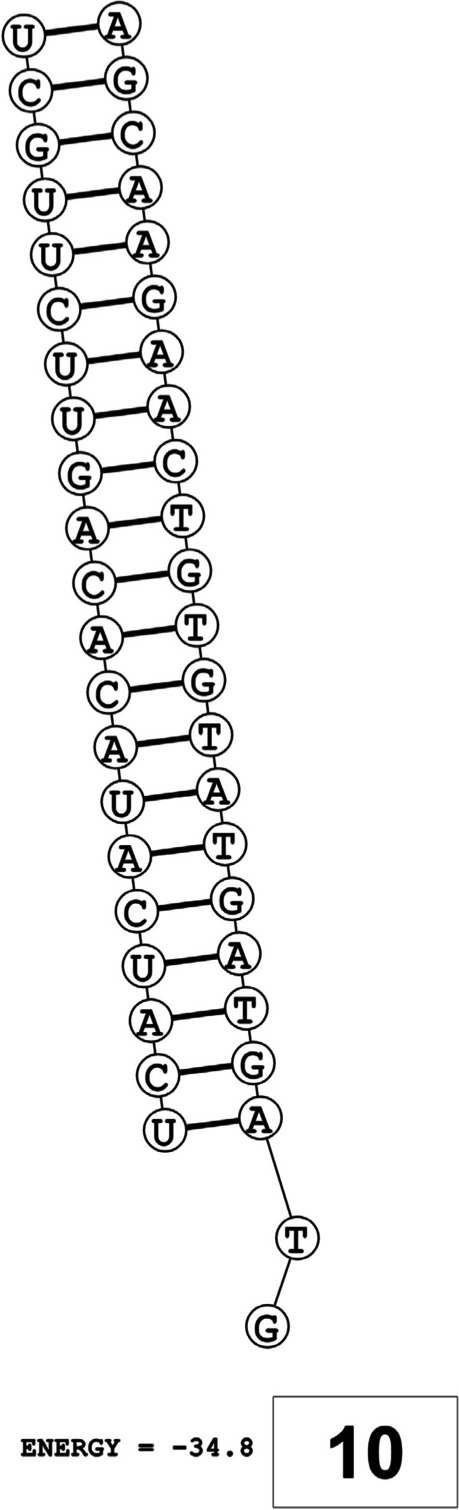
Secondary structure and MFE of siRNA-target duplex for NSP6 regions

**Fig. 16:**
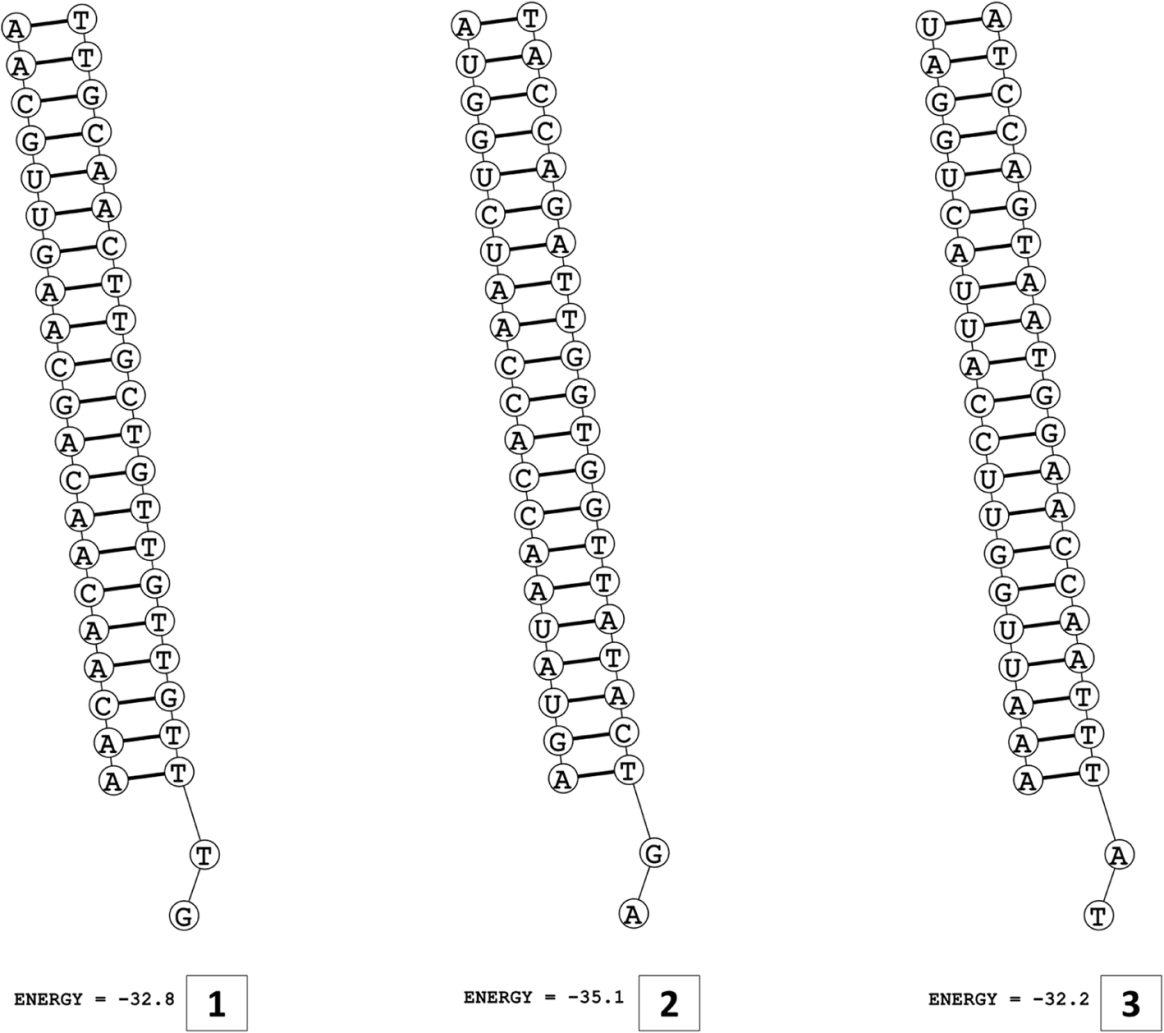
Secondary structures and MFE of siRNA-target duplexes for ORF3a regions

**Fig. 17:**
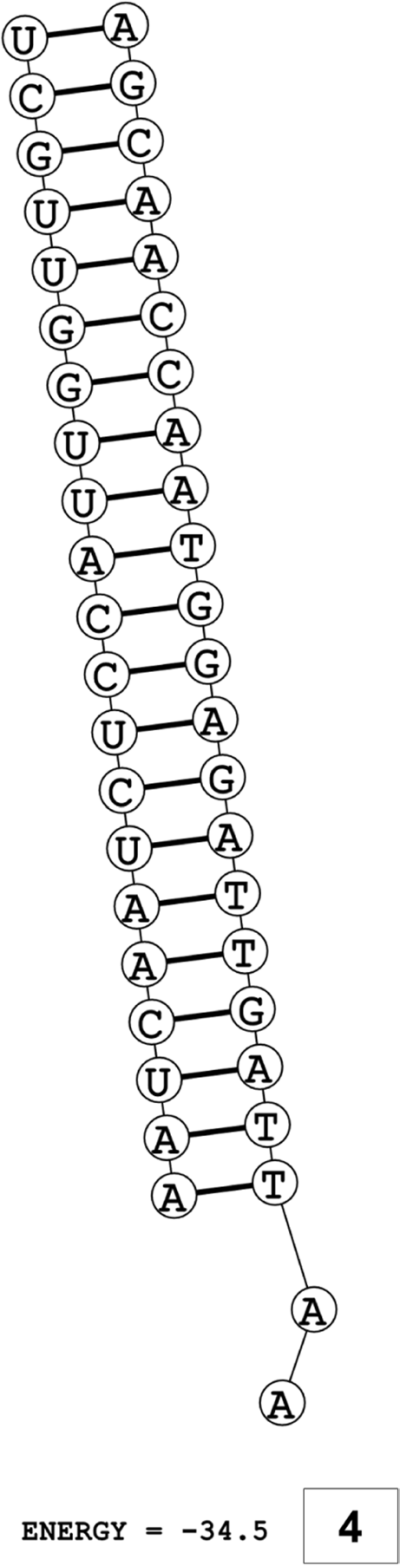
Secondary structures and MFE of siRNA-target duplex for ORF6 regions

**Fig. 18:**
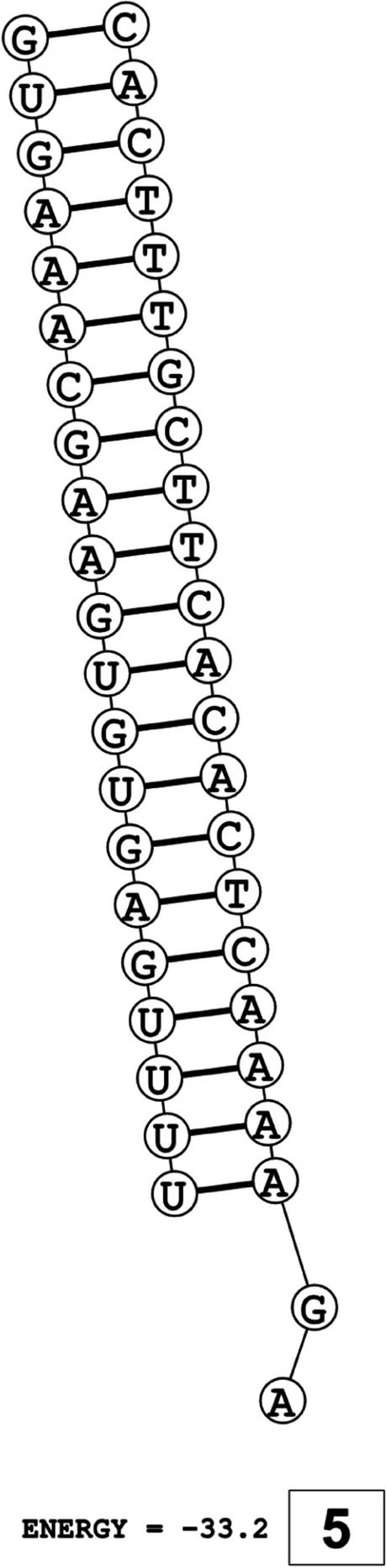
Secondary structure and MFE of siRNA-target duplex for ORF7a regions

**Fig. 19:**
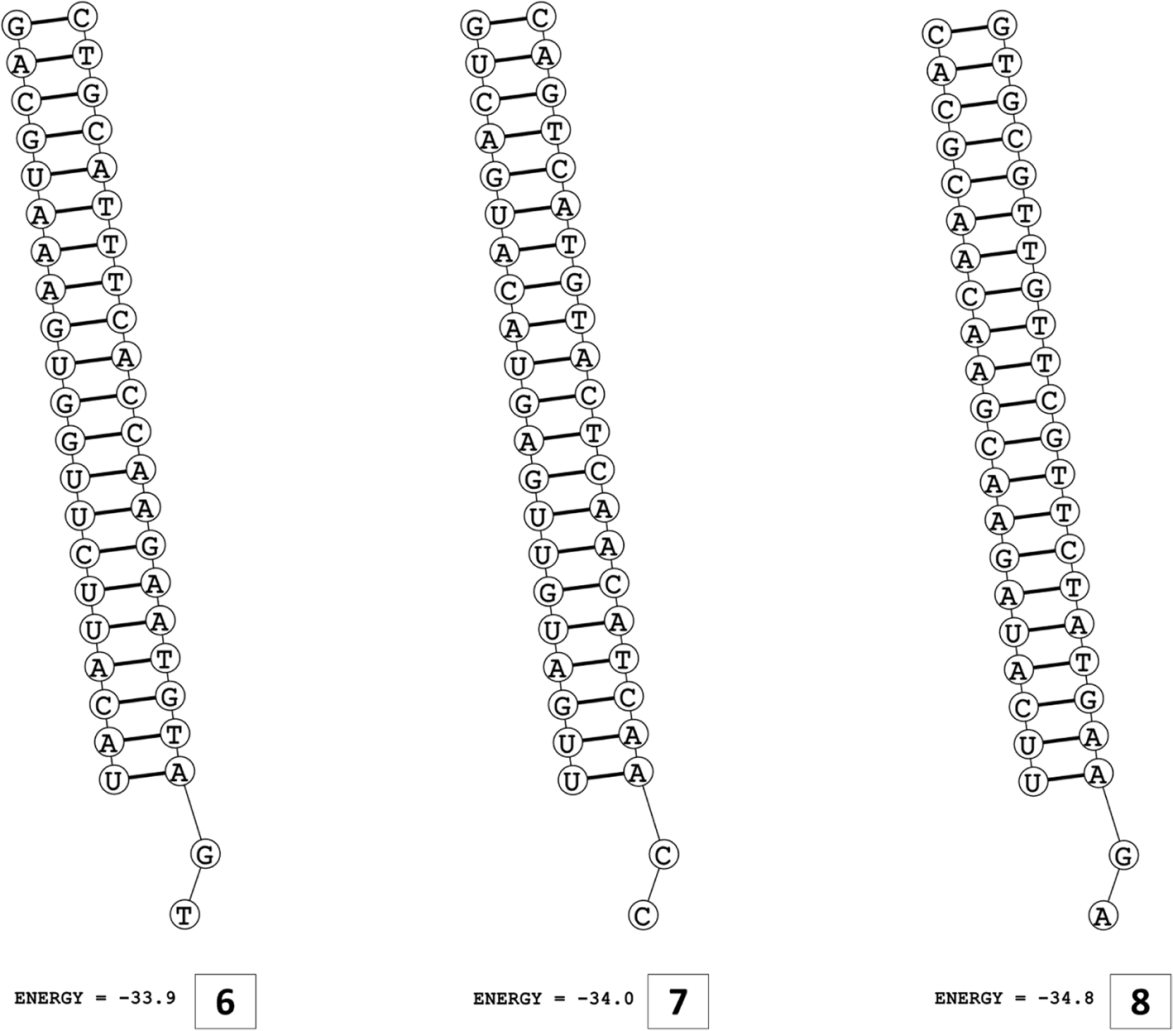
Secondary structures and MFE of siRNA-target duplexes for ORF8 regions

**Fig. 20:**
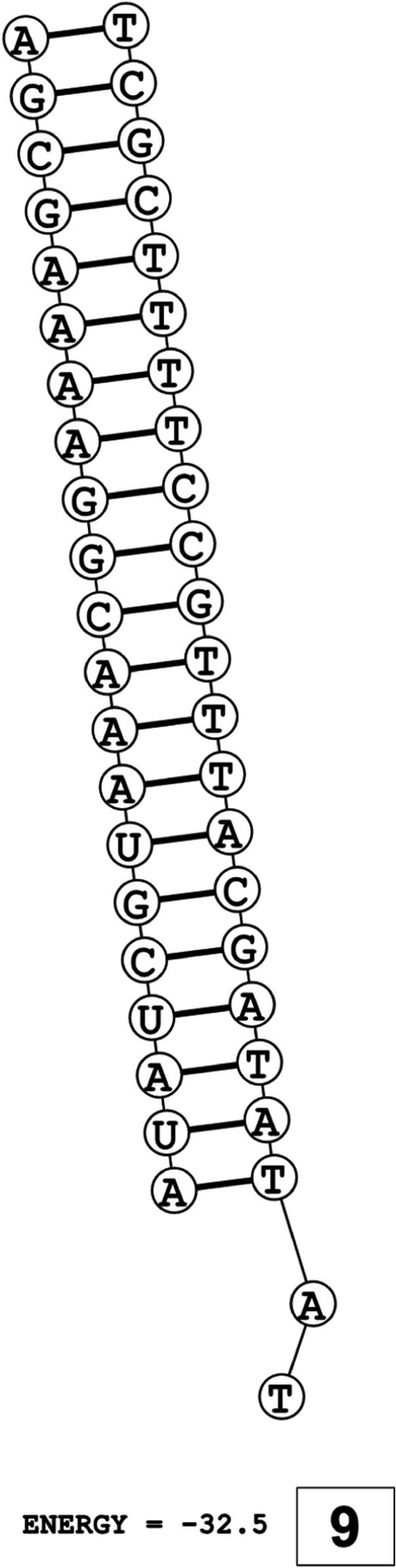
Secondary structure and MFE of siRNA-target duplex for ORF10 regions

The secondary structures of guide strands of the siRNA molecules targeting non-structural genes were predicted (Figs. 5, 6 and 7). The minimum free energy of folding ranged from 1.5 to 1.8 kcal/mol for non-structural genes (Table 1). For accessory genes, the secondary structures of folding were also predicted (Figs. 8, 9, 10, 11 and 12), and the minimum free energy values ranged from 1.4 to 1.9 kcal/mol (Table 2). The MFE value for one siRNA targeting ORF8 was found to be lower than the cutoff value (1.5 kcal/mol), thus, it was excluded from further study. The secondary structures of siRNA-target duplexes were also predicted for nonstructural (Fig. 13, 14 and 15) and accessory genes (Figs. 16, 17, 18, 19 and 20). The minimum free energy of binding ranged from − 36.8 to − 31.4 kcal/mol for non-structural genes (Table 1). For accessory genes, the minimum free energy of hybridization values ranged from − 35.7 to − 29.9 kcal/mol (Table 2). The MFE value for siRNA targeting ORF6 was found to be greater than the cutoff value (− 30 kcal/mol), thus, it was excluded from the study and further assessments.

### Tertiary structure prediction and validation

**Fig. 21:**
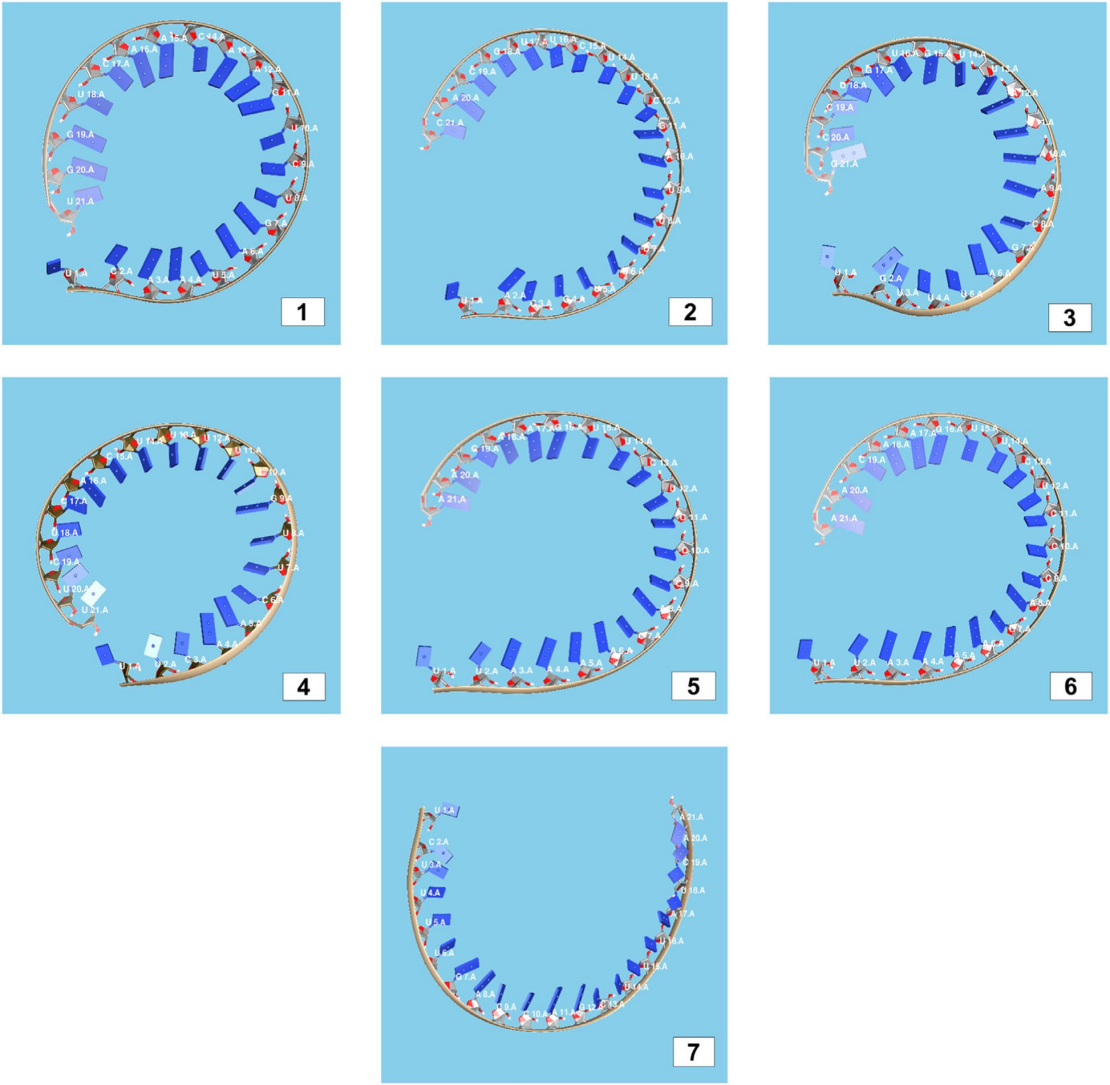
Tertiary structures of siRNA molecules targeting NSP3

**Fig. 22:**
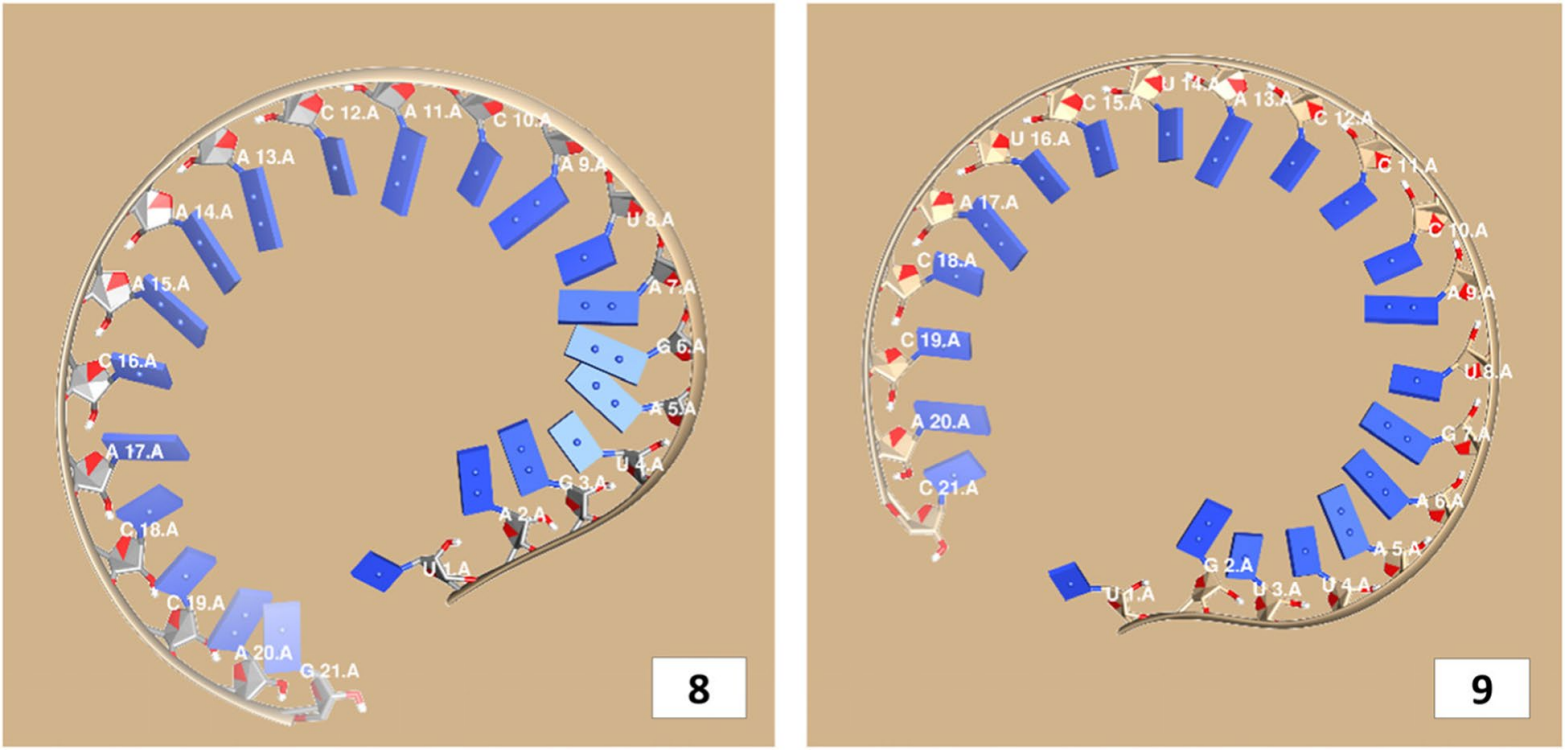
Tertiary structures of siRNA molecules targeting NSP4

**Fig. 23:**
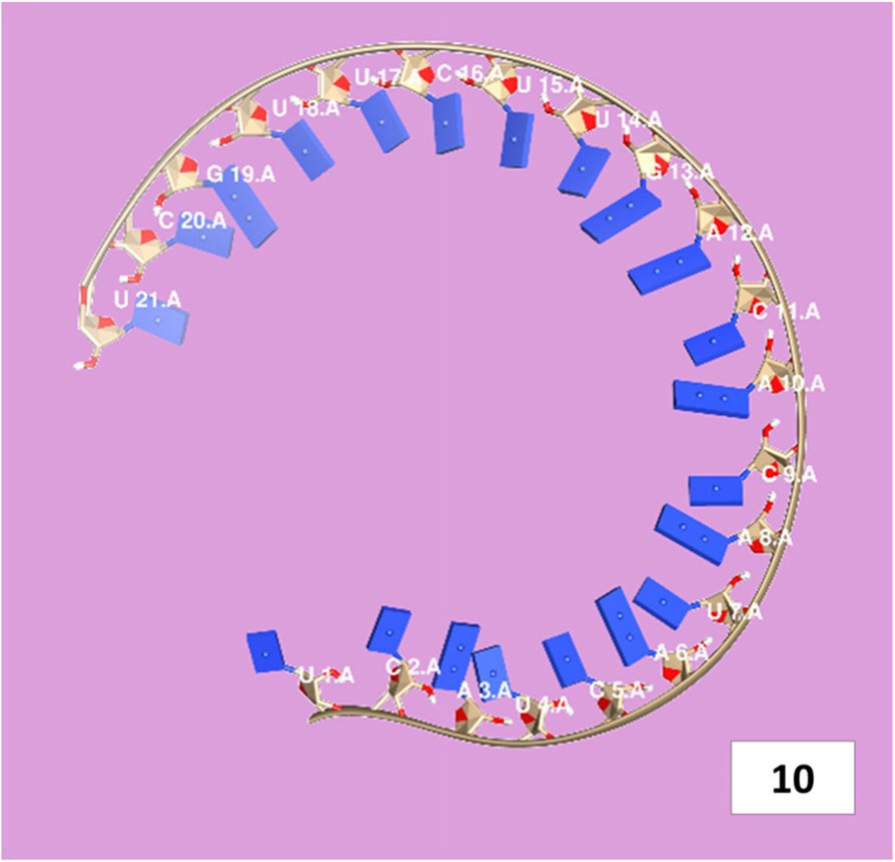
Tertiary structure of siRNA molecule targeting NSP6

**Fig. 24:**
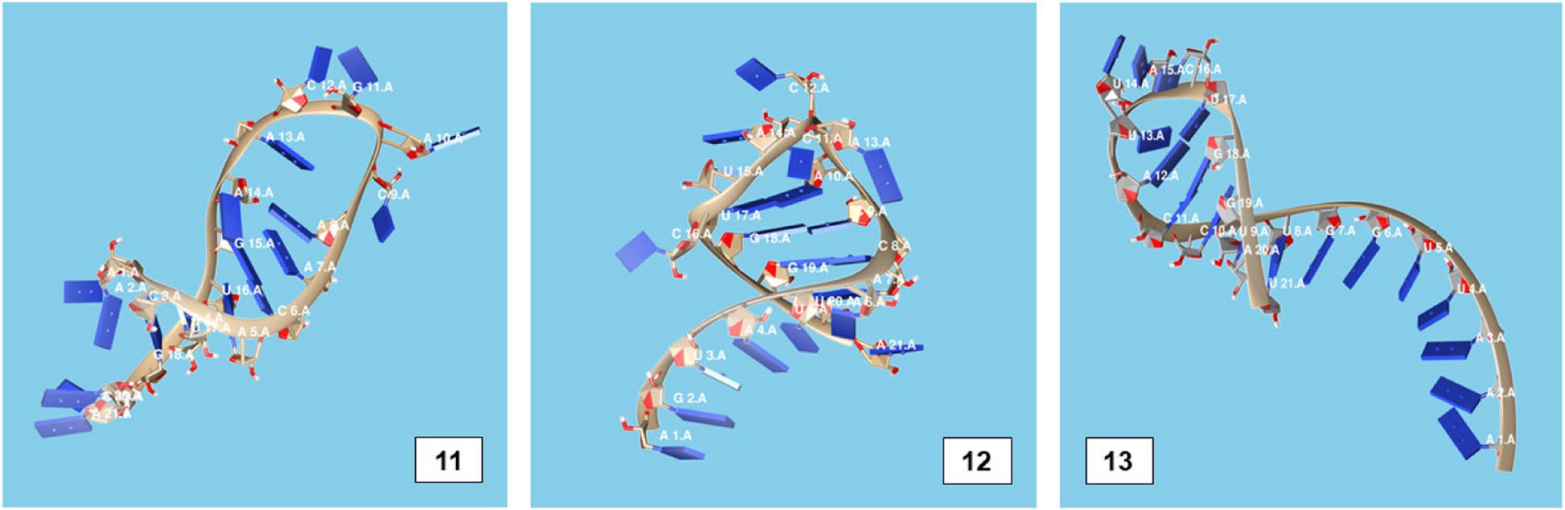
Tertiary structures of siRNA molecules targeting ORF3a

**Fig. 25:**
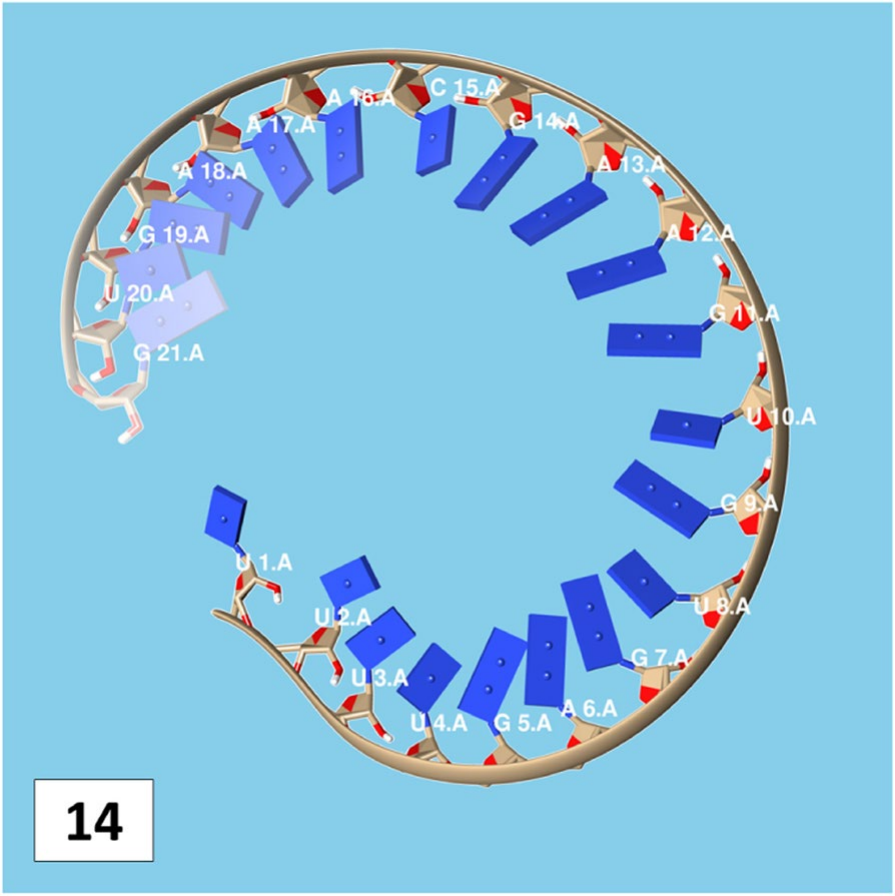
Tertiary structure of siRNA molecule targeting ORF7a

**Fig. 26:**
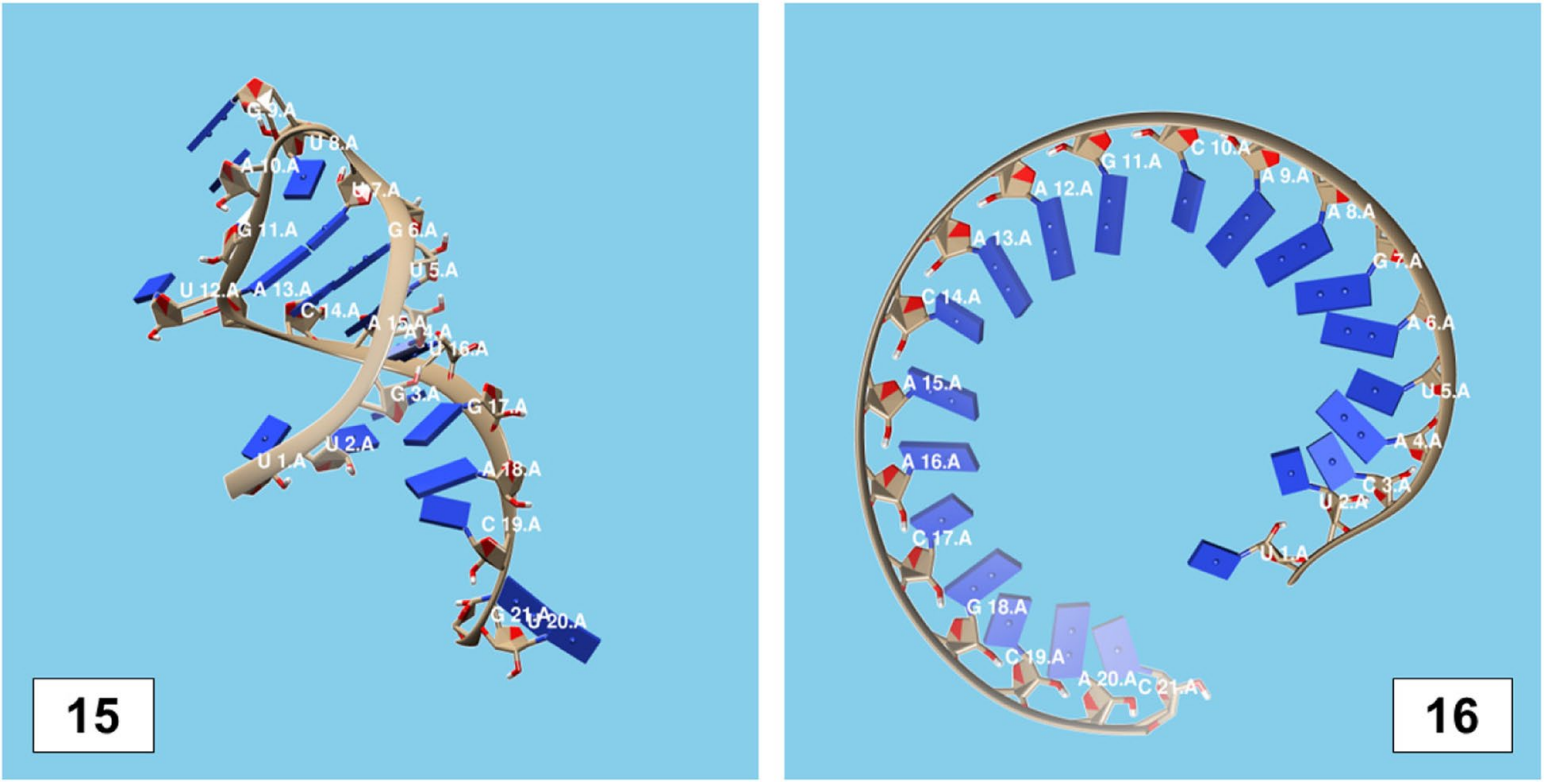
Tertiary structure of siRNA molecules targeting ORF8

**Fig. 27:**
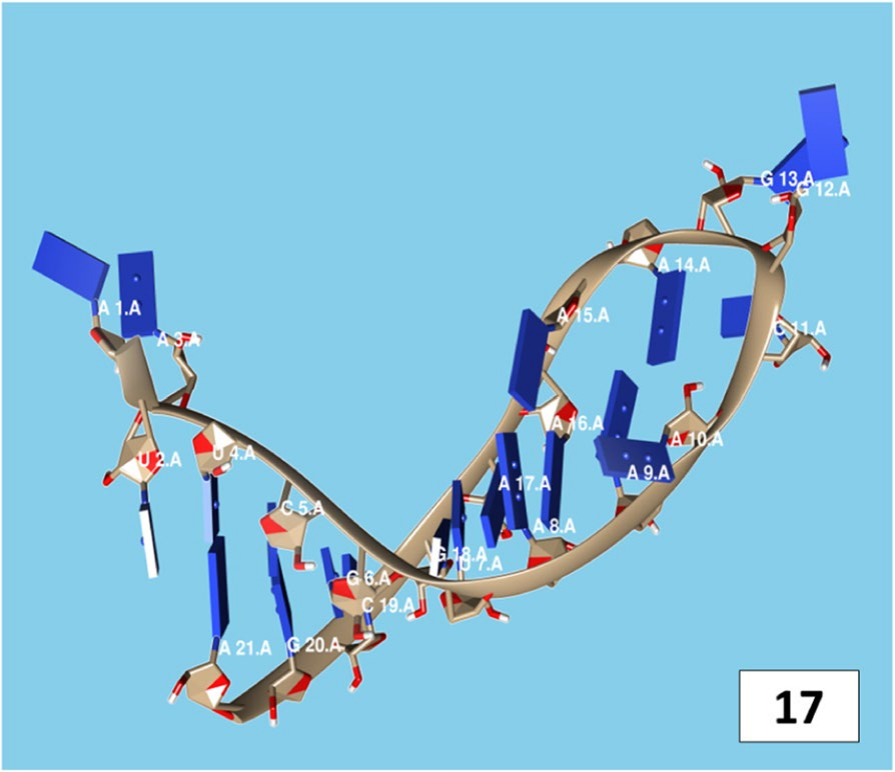
Tertiary structure of siRNA molecule targeting ORF10

The tertiary structures of guide strands of final 17 siRNA molecules, which passed the validation criteria were modelled and viewed using UCSF Chimera 1.16 (Figs. 21, 22, 23, 24, 25, 26 and 27). The 3D models obtained were further validated using MolProbity server (Additional file 1: Table S8). The nucleic acid geometry including probably wrong sugar puckers, bad backbone conformations, bad angles, bad bonds, and the chiral volume outliers for tertiary structures of siRNA molecules and additional validations were observed (Table 3).

**Table 3:** Nucleic acid geometry of siRNAs targeting non-structural and accessory genes of SARS-CoV-2: The tertiary structural validation of siRNA molecules against NSP3, NSP4 NSP6, ORF3a, ORF7a, ORF8, and ORF10 genes of SARS-CoV-2 using the MolProbity server

Target gene	NSP3							NSP4		NSP6	ORF3a			ORF7a	ORF8		ORF10
siRNA	1	2	3	4	5	6	7	8	9	10	11	12	13	14	15	16	17
**Probably wrong sugar puckers**	0	0	0	0	0	0	0	0	0	0	2*	2*	0	0	0	0	0
**Bad backbone conformations**	0 (0.00%)	0 (0.00%)	1 (4.76%)	0 (0.00%)	0 (0.00%)	1 (4.76%)	0 (0.00%)	1 (4.76%)	1 (4.76%)	2* (9.52%)	8* (38.10%)	6* (28.57%)	0 (0.00%)	0 (0.00%)	5* (23.81%)	1 (4.76%)	6* (28.57%)
**Bad bonds**	0/495 (0.00%)	0/471 (0.00%)	0/494 (0.00%)	0/471 (0.00%)	0/489 (0.00%)	0/493 (0.00%)	0/481 (0.00%)	0/500 (0.00%)	0/484 (0.00%)	0/481 (0.00%)	0/503 (0.00%)	0/494 (0.00%)	0/492 (0.00%)	0/504 (0.00%)	0/497 (0.00%)	0/497 (0.00%)	0/508 (0.00%)
**Bad angles**	0/769 (0.00%)	0/728 (0.00%)	0/768 (0.00%)	0/728 (0.00%)	0/758 (0.00%)	0/765 (0.00%)	0/745 (0.00%)	0/777 (0.00%)	0/750 (0.00%)	0/745 (0.00%)	0/782 (0.00%)	0/767 (0.00%)	0/764 (0.00%)	0/785 (0.00%)	0/773 (0.00%)	0/772 (0.00%)	0/791 (0.00%)
**Chiral volume outliers**	0/104	0/104	0/104	0/104	0/104	0/104	0/104	0/104	0/104	0/104	0/104	0/104	0/104	0/104	0/104	0/104	0/104

* The highlighted nucleic acid conformations (above the suggested threshold values) were detected in the tertiary structure of siRNAs, however, these percentages have not imposed any hinderance in the determination of siRNA-target hybridization at secondary structural level

### Off-target minimization

No off-target effects were found for target sequences of predicted siRNA molecules in BLASTn results against the Human Genomic + Transcript Database and the E-values were found to be all non-significant.

### Conservation analysis of designed siRNAs against SARS-CoV-2 variants

The target sequences of designed siRNA molecules were found to be highly conserved in the genome sequences of SARS-CoV-2 variants at each targeted position. This suggested that the designed siRNAs had capability to target the genome sequences of all SARS-CoV-2 variants of concern efficiently (Tables 1, 2).

## Discussion

When compared to the genomes of other RNA viruses, coronaviruses have been found to possess largest genome sizes. They are capable of establishing reservoirs in both human and zoonotic populations, enabling their transmission and circulation among a range of animal hosts, including bats, pangolins, civets, cats, mice, pigs, whales, dogs, and raccoons [39]. Till date, SARS-CoV-2 is regarded as the most lethal among the family of coronaviruses. The genome of SARS-CoV-2 consists of fourteen Open Reading Frames (ORFs), which encode for 16 non-structural proteins, 4 structural proteins and 11 accessory factors [7, 8]. The 2 major polyproteins, ORF1a and ORF1ab, are present in SARS-CoV-2 proteome, which are to produce individual replicase complex nonstructural proteins. These nonstructural proteins (NSP1–16), play a crucial role in regulating early transcription and facilitating genome replication [9]. NSP3, NSP4, and NSP6 are collectively involved in the formation and assembly of double membrane vesicles (DMVs) within the Golgi apparatus of the host. These DMVs provide a site for the anchorage of viral replication complexes, which facilitate viral genome replication and the production of progeny virions within the host cell upon their release, thus enabling further infection [10]. On the other hand, accessory genes of SARS-CoV-2 also play a major role in regulating replication and contribute in the pathogenicity of virus. Previous studies reported deletion of accessory genes ORF3a, 3b, 5a and 5b from avian coronavirus and observed resultant mutated virus exhibiting reduced pathogenicity [40]. Thus, targeting accessory genes of SARS-CoV-2 can be an effective strategy for therapeutic purposes. For this study, the sequences of non-structural (NSP3, 4, and 6) and accessory genes (ORF3a, 6, 7a, 8, and 10) were utilized to predict short interfering RNA molecules that could potentially interfere with SARS-CoV-2.

Sohrab et al., 2022 predicted 4 siRNAs for targeting the receptor binding domain (RBD-S) of SARS-CoV-2 using an in silico pipeline. They found no cytotoxicity in the Vero E6 cell line based experimental evaluation of the predicted siRNAs and one out of four siRNAs showed better antiviral activity based on qPCR Ct value [26]. In another study by Sohrab et al., 2021, they identified 7 efficient siRNA molecules for targeting ORF1ab of MERS-CoV using siDirect 2.0 and their designed siRNAs showed no cytotoxic effects in Vero cells (ATCC CCL-81) at different concentrations. They identified 2 out of 5 siRNAs for the inhibition of viral replication more efficiently on the basis of real-time PCR [25]. Perez-Mendez et al., 2021 also targeted the 5′ UTR region of Zika virus via an siRNA designed in silico. A significant reduction in cycle thresholds was found in C6/36 cells when transfection with 1 and 2 μg of the synthesized siRNA was done in infected cells at an MOI of 0.001 for one hour (*p <* 0.05) [23]. ElHefnawi et al., 2016 also predicted 2 siRNAs against 5′ NTR of Hepatitis C virus. Both of the siRNAs (HCV353 and HCV258) showed efficient inhibition of HCV replication mechanism at low concentrations. Moreover, both siRNAs suppressed the replication of HCV genotype 4 isolates derived from infected Huh-7 cells efficiently. The long-term treatment of HCV replicon cells also did not lead to the emergence of escape mutant viruses which ensured the sustained effectiveness of the antiviral therapy over an extended time period [19]. We developed a novel in silico pipeline for predicting and validating siRNA molecules that combines multiple effective in silico methods used in the previous studies [19, 23, 25, 26], which demonstrated successful inhibition of viral replication in vitro. This innovative pipeline confidently aims to identify and validate siRNAs with the potential to inhibit viral replication in in vitro experiments (Table 4).

**Table 4:** Comparative analysis of in silico pipelines from the previous antiviral siRNA studies and their in vitro implementation results

Targeted viruses and their genomic region	In silico siRNA designed pipeline												Total designed siRNAs	In vitro implementation and results
	Sequence retrieval from repositories & databases	Multiple Sequence Alignment	Consensus sequence generation	Phylogenetic analysis	siRNA prediction using siDirect 2.0	Binary validation using siRNApred	i-Score and s-Biopredsi scores calculation	Thermo-dynamic stability	Secondary structures prediction & MFE calculation using RNAStructure	Tertiary structure Prediction using RNA Composer	Molprobity validation	Off-target minimization		
Receptor-binding domain (RBD-S) of SARS-CoV-2 [26]	✓^a^	✘	✘	✘	✓	✘	✘	✘	✘	✘	✘	✘	4	No cytotoxicity for tested siRNAs was found in Vero E6 cells based on experimental evaluation and analysis of generated results. Following strict selection and scoring criteria, a better antiviral efficiency was observed in 1 out of 4 siRNAs based on q-real-time PCR Ct value.
ORF1ab of MERS-CoV [25]	✓^a^	✓	✘	✘	✘	✘	✓	✘	✘	✘	✘	✘	7	siRNAs showed no cytotoxic effects at various concentrations in Vero cells (ATCC CCL-81). On the basis of real-time PCR results, two of the designed siRNAs were found to inhibit viral replication more efficiently as compared to the other five.
5′ UTR of Zika virus [23]	✓^a^	✓	✓	✓	✓	✘	✘	✘	✓	✓	✓	✓	1	Significant reduction in cycle thresholds was observed in C6/36 cells upon transfection with 1 and 2 μg of designed siRNA after being infected with Zika virus at an MOI of 0.001 for 1 hour (*p <* 0.05).
5′ NTR of Hepatitis C virus [19]	✓^b^	✓	✓	✘	✘	✘	✓	✘	✘	✘	✘	✓	2	Both of the designed siRNAs (HCV353 and HCV258) demonstrated efficient inhibition of HCV replication mechanism at low concentrations. Moreover, both siRNAs suppressed the replication of HCV genotype 4 isolates derived from infected Huh-7 cells efficiently. Also the long-term treatment of HCV replicon cells did not result in the emergence of escape mutant viruses.

^a^ Retrieved from NCBI

^b^ Retrieved from HCV LANL

Multiple sequence alignment of selected gene sequences was performed for the conservation analysis. The sequences of NSP3, NSP4, and NSP6 showed high levels of conservation among the 100 selected sequences of each gene in the circulating strains of SARS-CoV-2 across the globe, from year 2019 to 2023. It was also observed that the NSP3 sequences exhibited a substantial frequency of mutations. Our observation is consistent with a previous study on conservation and mutational analysis of nonstructural genes of SARS-CoV-2, on the basis of geographic distribution by Anand et al., 2021, in which some of the highly mutating positions in NSP3 were reported as “hotspot zones” [41]. In another conservation and phylogenetic analysis by Fiaz et al., 2021, NSP3 was reported as the most variable nonstructural gene [42]. Among our target sequences, a point mutation was observed in NSP3 sequence of a Japanese strain (accession number = OQ504245.1) showing Guanine in place of highly conserved Adenine residues at position 1140. Another mutation was observed in NSP4 sequences, showing Thymine in place of conserved Cytosine residues at position 732, in a strain from Switzerland (accession number = OQ050229.1). Our conservation analysis of accessory genes also revealed a high level of conservation among the selected sequences. In a previous conservation analysis of accessory proteins of SARS-CoV-2, Li et al., 2020 reported diverse mutations disseminated within ORF3a and ORF8 [43].

The phylogenetic analyses demonstrated variability across various geographic regions and revealed multiple clades with distinct clusters. In phylogenetic tree constructed for NSP3 sequences, the clusters A, C, D, and J showed a uniform distribution of Asian and European sequences predominantly. Among other obtained clusters, NSP3 sequences of Pakistani strains from years 2022 and 2023 fall in clusters D, F, and I with Asian, European, and African sequences. Overall, phylogenetic analysis of NSP3 sequences revealed highest rate of variations. In a previous genomic and epidemiological study, Lamptey et al., 2021 also performed phylogenetic analyses of nonstructural proteins of SARS-CoV-2 and found that NSP3 sequences contained most variants [44]. The phylogenetic analysis of NSP4 also revealed the same distribution pattern of sequences from different continents across various obtained clusters. Predominantly, most of the European sequences were found in cluster A (*n* = 16/30) along with Asian sequences (*n* = 8/30). Cluster B contained sequences from New Zealand strains of 2021 and 2022 sharing close relatedness with US strains. Asian strains were found to be predominant in clusters F and G also, along with a uniform distribution of sequences from Europe and other continents. The Pakistani sequences of NSP4 fell in clusters A and G sharing close relatedness with European, Asian, and US sequences. The phylogenetic analysis of NSP6 gene from circulating strains across the globe revealed a uniform distribution of sequences throughout the phylogenetic tree. The phylogenetic analysis of accessory gene revealed high levels of conservation and the sequences were uniformly distributed throughout the respective clusters. Further sequence logo analyses were performed and consensus sequences were obtained using WebLogo application and Jalview program respectively.

Short interfering RNAs are small (21 to 25 nt) RNA molecules that do not encode for proteins and have the ability to bind to complementary messenger RNA sequences. At post-transcriptional level, they can prevent the mRNA from being translated into proteins, thereby negatively regulating the expression of the target gene. An siRNA requires a high degree of complementarity between the guide strand of the siRNA and its specific target mRNA. Since the discovery of siRNA therapy, significant advancements have been made in investigating the potential of small interfering RNA (siRNA) as a therapeutic approach for targeting genes of various viruses including Zika virus [23], Hepatitis C virus [19], Nipah virus [22], Influenza A virus [21], MERS-CoV [24], and SARS-CoV-2 [16, 17]. The web-based siDirect 2.0 [31] server employs a highly efficient algorithm and combined rational rules of Ui-Tei along with Reynolds + Amarzguioui for the prediction of functional siRNAs with minimal off-target effects. These rules design siRNAs having A or U residues at the 5′ end of guide strand. The guide strands with these thermodynamically unstable 5′ ends contribute strongly to the incorporation of siRNA into RISC complex and binding with Argonaute (Ago2) protein. The Tm value of 21.5 °C can be used as a threshold to distinguish the seed sequences with minimized off-target effects from those that are likely to have off-target binding effects. Primarily, a total of 41, 12, and 3 siRNA molecules were predicted against NSP3, NSP4, and NSP6 genes respectively and 7, 1, 2, 4, and 1 siRNAs were predicted for targeting regions of ORF3a, ORF6, ORF7a, ORF8, and ORF10 and further comprehensive analyses were performed, taking into consideration various filters to evaluate their effectiveness.

The GC content of siRNA-target duplexes is one of the significant parameters that may affect the efficacy of siRNA. A higher GC content may lead to the formation of secondary structures like hairpins and stems, which can ultimately lead to reduced accessibility of siRNA to its mRNA target. A lower GC content may result in an unstable duplex formation reducing the gene silencing efficiency. Therefore, in our study, an optimal GC content range of 31.6 to 57.0% was set to design efficient siRNAs. The predicted siRNA sequences were screened against the Main21 dataset of siRNAPred server using binary pattern [32]. Based on the highest binary scores (≥0.9), a total of 12, 2, and 1 siRNAs for NSP3, NSP4, and NSP6 respectively, and in case accessory genes, a total of 3, 1, 1, 3, and 1 siRNAs against ORF3a, 6, 7a, 8, and 10 respectively, were selected for the additional assessment. Further scoring of siRNA molecules was performed using i-Score Designer server that employs several 1st and 2nd generation algorithms [33]. The i-Scores (≥65) and s-Biopredsi scores (< 1) were calculated for evaluation of specificity of predicted siRNA sequences. In the heat capacity plots, C_p_ is represented as a function of temperature, referred to as T_m_C_p_. Whereas, T_m_ (Conc) represents the point at which concentrations of the siRNA-duplexes reach ½ of their maximum value. The melting temperatures T_m_C_p_ and Tm were calculated using DINAMelt server [34]. In case of non-structural genes, the T_m_C_p_ values ranged from 81.0 to 85.8 °C whereas the T_m_ (conc) values ranged from 79.6 to 84.5 °C. For accessory genes, the T_m_C_p_ values ranged from 81.3 to 86.2 °C whereas the T_m_ (conc) values ranged from 80.2 to 84.6 °C. For the visualization of folding and binding patterns along with their corresponding minimum free energy values, RNA structure program [35] was utilized. The secondary structures of guide strands of siRNA molecules were predicted using MaxExpect algorithm and their minimum free energy values ranged from 1.5 to 1.8 kcal/mol for NSPs and 1.4 to 1.9 kcal/mol for accessory genes. According to Hasan et al., 2021, positive MFE value indicates better siRNA molecules, as chances of folding are rare among them [17]. The secondary structures of target-siRNA duplexes were also predicted using RNA DuplexFold algorithm and the free energy of hybridization with target sequences of predicted potential siRNAs were − 34.8, − 33.9, − 35.0, − 31.4, − 31.5, − 32.5, − 31.9, − 34.2, − 36.8, and − 34.8 kcal/mol respectively. On the other hand, for accessory genes, the hybridization of siRNA-target mRNA duplex along with minimum free energy (MFE) for binding of both strands were − 32.9, − 35.7, − 32.2 kcal/mol for ORF 3a, − 29.9 for ORF6, − 33.2 kcal/mol for ORF7a, − 34.0, − 33.9, − 32.2 kcal/mol for ORF 8, and − 32.5 kcal/mol for ORF10. The MFE for an siRNA targeting ORF8 was found to be lower than the threshold value (1.5 kcal/mol), therefore, it was excluded from further analyses. Similarly, Minimum free energy of binding for siRNA targeting ORF6 was found to be greater than cutoff value (− 30 kcal/mol), thus, it was also excluded from further assessments. Next, we predicted the tertiary structures of 17 siRNA molecules using RNAComposer web server [36]. The chemical structure of RNA backbone is rotameric, and there is a probability of getting nucleic acid geometry below or above the suggested threshold values [45]. In order to validate the three-dimensional structures and nucleic acid geometry of our modelled siRNAs, we screened them using the MolProbity server [37].

siRNA enters the cell and come in contact with RNAi silencing machinery referred to as RNA induced silencing complex (RISC). Guide strand then attaches itself with this complex leaving the passenger strand, which is then removed. It causes the attachment of this complex with a protein namely, argonaute thereby activating the complex. Guide strand directs this complex with its target mRNA sequence and binding occurs. Out of 21 nucleotides of siRNA, 19 of them acts as recognition factor for the silencing of gene by its breakdown [46]. The nucleotides present at position 2–8 are termed as seed region which should not be complementary to any nontargeted mRNA sequence to prevent off target effects [47]. Therefore, finally we performed nucleotide BLAST [28] (BLASTn) against human genomic plus transcript database for investigation of any off-target effects and found no significant E-values.

In a previous study conducted by Saadat et al., 2022, a total of 133 siRNA molecules were predicted against a number of targeted proteins including non-structural and structural proteins and the 5′ and 3′ UTR sequences of SARS-CoV-2 [48]. They have reported 45 siRNA molecules for targeting NSP3/PLpro using siDirect 2.0, however, no siRNA candidate shared sequence similarity with our predicted siRNAs. In another study, Hasan et al. 2021 reported a total of 10 siRNA molecules, predicted against ORF1ab of SARS-CoV-2 using the same tool [17]. Our study, on the other hand, was focused on predicting siRNAs for NSP3, NSP4, and NSP6 of SARS-CoV-2 and identified 10 potential siRNA molecules. Among these, 3 siRNA molecules targeting NSP3 (siRNA no. 1, 2, and 3) were found to have complete sequence similarity with the siRNAs predicted by Hasan et al. 2021, thus validating our findings. Additional in vitro and in vivo experiments are needed to validate the effectiveness and role of the predicted siRNAs in suppressing NSP3, 4, and 6 along with accessory genes for inhibiting the double membrane vesicle formation and replicative pathway of SARS-CoV-2.

## Conclusion

siRNAs (short interfering RNAs) are a promising approach for treatment of a number of viral infections by targeting conserved regions of viral genomes. In this in silico methodology, we conducted a conservation analysis of the three non-structural genes of SARS-CoV-2, which participate in formation of double membrane vesicles (DMVs), as well as the viral accessory genes. A total of 17 highly specific and potential candidate siRNAs were selected after passing a number of filters and validation criteria including 7, 2, and 1 siRNA molecules against NSP3, NSP4, and NSP6, and 3, 1, 2, and 1 siRNAs against ORF3a, ORF7a, ORF8, and ORF10 respectively. We designed a unique in silico pipeline for predicting and validating siRNA molecules based on multiple effective pipelines used for designing siRNAs in previous studies demonstrating successful in vitro inhibition of viral replication. This computational study might prove useful for development of an effective antiviral therapy for inhibiting viral replication and might prove to be an additional reputed intervention in life threatening conditions.

### Supplementary Information

**Additional file 1.** Supplementary Tables

## Data Availability

The datasets supporting the conclusion of this article are included within its Additional file 1.

## Abbreviations

COVID-19: Coronavirus disease of 2019
C_p_: Heat Capacity
DMV: Double membrane vesicle
ncRNA: Non-coding RNA
NSP: Non-structural protein
ORF: Open reading frame
RNAi: RNA interference
siRNA: Short interfering RNA
SARS-CoV-2: Severe acute respiratory syndrome coronavirus 2
T_m_: Melting Temperature

## Footnotes

1 https://www.ncbi.nlm.nih.gov/labs/virus/vssi/#/

2 https://www.ncbi.nlm.nih.gov/orffinder/

3 https://weblogo.berkeley.edu/logo.cgi

4 http://sidirect2.rnai.jp/

5 http://www.endmemo.com/bio/gc.php

6 http://crdd.osdd.net/raghava/sirnapred/

7 https://www.med.nagoya-u.ac.jp/neurogenetics/i_Score/i_score.html

8 http://www.unafold.org/hybrid2.php

9 https://rnacomposer.cs.put.poznan.pl/

10 http://molprobity.manchester.ac.uk/

11 http://crdd.osdd.net/raghava/sirnapred/
