# Supplementary material for: An ncRNA transcriptomics-based approach to design siRNA molecules against SARS-CoV-2 double membrane vesicle formation and accessory genes
Source: BMC Infect Dis. 2023 Dec 12;23:872. doi: 10.1186/s12879-023-08870-0 (PMC10718025; doi:10.1186/s12879-023-08870-0)
Supplement: Supplementary file 1 — Additional file 1. Supplementary Tables [file 12879_2023_8870_MOESM1_ESM.docx]

**Additional file 1: Supplementary Tables**

| **Table S1.** Accessions numbers of selected SARS-CoV-2 genome sequences from Australia, Austria, Bangladesh, Brazil, California, Canada, China, Colombia, Egypt, Finland, Florida, France, Germany, Hong Kong, India, Italy, Japan, Malaysia, Mexico, New York, New Zealand, Nigeria, Norway, Pakistan, Russia, South Africa, Spain, Sweden, Switzerland and Turkey. | | | | |
| --- | --- | --- | --- | --- |
| **Australia** | **Austria** | **Bangladesh** | **Brazil** | **California** |
| MW320915.1 | OQ520264.1 | ON974872.1 | OP898570.1 | ON825124.1 |
| ON532671.1 | ON112351.1 | ON909199.1 | OQ521671.1 | OP202480.1 |
| OP604177.2 | OQ520264.1 |  |  | OP402670.1 |
|  | OM283602.1 |  |  | OP943924.1 |
|  |  |  |  | OQ546924.1 |
| **Canada** | **China** | **Colombia** | **Egypt** | **Finland** |
| OM367886.1 | OP804248.1 | OP303166.1 | OP183416.1 | OP435368.1 |
| OM540774.1 | OQ372907.1 | OP303171.1 | OP183453.1 | OQ411059.1 |
| ON239776.1 | OQ179919.1 | OP303175.1 | OL351370.1 |  |
| OP237527.1 | OQ170935.1 | OP303177.1 |  |  |
|  |  |  |  |  |
| **Florida** | **France** | **Germany** | **Hong Kong** | **India** |
| OQ520413.1 | OQ423333.1 | OQ503465.1 | ON599967.1 | OP295728.1 |
| OQ533737.1 | OP646526.1 | ON003597.1 | OP550452.1 | OP800207.1 |
| OQ543309.1 | OP646493.1 | OP430881.1 |  | OQ288261.1 |
|  |  | OQ242011.1 |  | OP781960.1 |
|  |  | OQ332762.1 |  |  |
| **Italy** | **Japan** | **Malaysia** | **Mexico** | **New York** |
| OL738656.1 | BS007024.1 | ON652348.1 | ON148330.1 | OM082826.1 |
|  | BS007015.1 | OM995898.1 | OQ084428.1 | OQ520386.1 |
|  | BS004661.1 | ON527304.1 | OQ084190.1 | OP944713.1 |
|  | BS001762.1 |  | ON679528.1 | OP662348.1 |
|  | OQ504245.1 |  |  |  |
| **New Zealand** | **Nigeria** | **Norway** | **Pakistan** | **Russia** |
| ON669310.1 | OQ050243.1 | OM616037.1 | OP872677.1 | OQ318441.1 |
| OP631683.1 | OQ052530.1 | ON222956.1 | OQ244526.1 | OQ363227.1 |
| OP633568.1 |  |  | OQ519925.1 | OP810428.1 |
| OP719787.1 |  |  | OQ519930.1 |  |
| OP720005.1 |  |  | MZ328026.1 |  |
| **Spain** | **South Africa** | **Sweden** | **Switzerland** | **Turkey** |
| OL989058.1 | OM765525.1 | OP585708.1 | ON209723.1 | OM295705.1 |
| MZ914594.1 | OP053471.1 | OP585707.1 | ON159867.1 | OL549281.1 |
| MZ099821.1 | OQ341811.1 |  | OL689430.1 |  |
| MW769725.1 |  |  | OQ050229.1 |  |
| MZ901915.1 |  |  |  |  |

| **Table S2.** Accessions numbers of reference genome and variants of concern of SARS-CoV-2 | |
| --- | --- |
| **Variant/Sequence** | **Accession Number** |
| RefSeq | NC_045512.2 |
| Alpha (B.1.1.7) | OW998408.1 |
| Beta (B.1.351) | OX008586.1 |
| Gamma (P.1) | OX000832.1 |
| Delta (B.1.617.2) | OW998779.1 |
| Eta (B.1.525) | OX014268.1 |
| Kappa (B.1.617.1) | MZ157006.1 |
| Mu (B.1.621) | PA544053.1 |
| Zeta (P.2) | OW996226.1 |
| Omicron (BA.1) | OX315743.1 |
| RefSeq Reference sequence | |

| **Table S3.** Accessions numbers of selected SARS-CoV-2 genome sequences from, Austria, Brazil, Germany, Hong Kong, India, Korea, Libya New Zealand, Saudi Arabia, Switzerland, and USA. | | | | |
| --- | --- | --- | --- | --- |
| **Sr. no.** | **Accession Number** | **Host** | **Year** | **Location** |
| 1 | MW565625.1 | Human | 2020 | USA |
| 2 | MW495017.1 | Human | 2020 | Brazil |
| 3 | MZ331947.1 | Human | 2020 | New Zealand |
| 4 | OL675863.1 | Human | 2020 | Switzerland |
| 5 | OM640071.1 | Human | 2020 | Austria |
| 6 | MT582499.1 | Human | 2020 | Germany |
| 7 | MT630431.1 | Human | 2020 | Saudi Arabia |
| 8 | MT810119.1 | Human | 2020 | Korea |
| 9 | MT628700.1 | Feline | 2020 | Hong Kong |
| 10 | MT628701.1 | Human | 2021 | Libya |
| 11 | MZ054892.1 | Human | 2020 | India |
| 12 | MT434757.2 | Human | 2021 | USA |
| 13 | OL515307 | Human | 2021 | USA |
| 14 | MW888068 | Human | 2021 | USA |
| 15 | OK664057 | Human | 2021 | USA |
| 16 | OL516117 | Human | 2021 | USA |
| 17 | MZ194280 | Human | 2021 | USA |

| **Table S4.** Target position, target sequence, RNA oligo sequences, seed-duplex stability (Tm), GC content, and binary validity scores for predicted siRNA molecules for targeting NSP3 gene of SARS-CoV-2. | | | | | | |
| --- | --- | --- | --- | --- | --- | --- |
| **No.** | **Target position** | **RNA oligo sequences**  **21nt guide (5′→3′)**  **21nt passenger (5′→3′)** | **Seed-duplex stabilty (Tm)** | | **GC%** | **Binary Validity** |
|  |  |  | **Guide** | **Passenger** |  |  |
|  |  |  | **°C** | **°C** |  |  |
| **1** | 243-265 | UAGUAUGUAGCCAUACUCCAC  GGAGUAUGGCUACAUACUACU | 19.0 °C | 20.3 °C | 42.9 | 1.064 |
| **2** | 351-373 | UCAAACUCUUCUUCUUCACAA  GUGAAGAAGAAGAGUUUGAGC | 19.2 °C | 20.4 °C | 38.1 | 0.913 |
| **3** | 466-488 | UAACCAAUCUUCUUCUUGCUC  GCAAGAAGAAGAUUGGUUAGA | 18.8 °C | 16.6 °C | 38.1 | 0.968 |
| **4** | 530-552 | UAGUAGUUGUCUGAUUGUCCU  GACAAUCAGACAACUACUAUU | 17.6 °C | 20.5 °C | 35.7 | 0.900 |
| **5** | 597-619 | UCAAUAGUCUGAACAACUGGU  CAGUUGUUCAGACUAUUGAAG | 11.6 °C | 17.8 °C | 38.1 | 1.008 |
| **6** | 682-704 | UACCUUUUUAGCUUCUUCCAC GGAAGAAGCUAAAAAGGUAAA | 17.3 °C | 19.1 °C | 35.7 | 1.062 |
| **7** | 864-886 | UGUUUAGCAAGAUUGUGUCCG GACACAAUCUUGCUAAACACU | 20.9 °C | 19.3 °C | 40.5 | 1.072 |
| **8** | 971-993 | AUAAUGGUGCAAGUAGAACUU GUUCUACUUGCACCAUUAUUA | 20.0 °C | 18.9 °C | 33.33 | 0.951 |
| **9** | 1126-1148 | UUCAACUUGCUUUUCACUCUU GAGUGAAAAGCAAGUUGAACA | 19.2 °C | 19.2 °C | 35.7 | 1.030 |
| **10** | 1281-1303 | UUUUCUGUGAGGAACUUAGUU CUAAGUUCCUCACAGAAAACU | 19.2 °C | 14.6 °C | 35.7 | 0.968 |
| **11** | 1350-1372 | UCAAUGUCACUAACAAGAGUG CUCUUGUUAGUGACAUUGACA | 20.5 °C | 19.2 °C | 38.1 | 0.892 |
| **12** | 1415-1437 | UUAAAACACCCUCUUGAACAA GUUCAAGAGGGUGUUUUAACU | 7.2 °C | 20.4 °C | 35.7 | 1.031 |
| **13** | 1568-1590 | UUUUAAGCACUGUCUUUGCCU GCAAAGACAGUGCUUAAAAAG | 13.7 °C | 19.2 °C | 38.1 | 0.977 |
| **14** | 1830-1852 | AUAAGUGACGCUACAGUUGUU CAACUGUAGCGUCACUUAUCA | 20.3 °C | 19.0 °C | 40.5 | 0.947 |
| **15** | 2025-2047 | UGUUCUUCAGGUGUUUUAGAA CUAAAACACCUGAAGAACAUU | 21.5 °C | 7.2 °C | 33.33 | 1.017 |
| **16** | 2478-2500 | UUUAAUGCUGACAUGUACCUA GGUACAUGUCAGCAUUAAAUC | 15.3 °C | 20.4 °C | 35.7 | 1.079 |
| **17** | 2511-2533 | UUAACUUGUGGGUAUUUCCAC GGAAAUACCCACAAGUUAAUG | 11.8 °C | 10.0 °C | 38.1 | 1.029 |
| **18** | 2775-2797 | ACGUUCAAGACUCUUUUGCAA  GCAAAAGAGUCUUGAACGUGG | 21.1 °C | 12.2 °C | 42.9 | 0.978 |
| **19** | 2835-2857 | UACAUAACAGCUUCUACACCC  GUGUAGAAGCUGUUAUGUACA | 14.6 °C | 20.3 °C | 40.5 | 0.990 |
| **20** | 3084-3106 | AGUAAAGCACCGUCUAUGCAA  GCAUAGACGGUGCUUUACUUA | 20.9 °C | 20.3 °C | 42.9 | 0.930 |
| **21** | 3085-3107 | AAGUAAAGCACCGUCUAUGCA  CAUAGACGGUGCUUUACUUAC | 9.8 °C | 20.9 °C | 42.9 | 0.995 |
| **22** | 3291-3313 | UUUGGAUAUGGUUGGUUUGGU  CAAACCAACCAUAUCCAAACG | 21.2 °C | 18.8 °C | 40.5 | 0.945 |
| **23** | 3589-3611 | UGUUUCAACUGGUUUUGUGCU  CACAAAACCAGUUGAAACAUC | 14.9 °C | 13.3 °C | 38.1 | 0.957 |
| **24** | 3692-3714 | UAGGAUUUUCCACUACUUCUU  GAAGUAGUGGAAAAUCCUACC | 18.7 °C | 17.6 °C | 38.1 | 0.941 |
| **25** | 3752-3774 | UAAUGUCUCCUACAACUUCGG  GAAGUUGUAGGAGACAUUAUA | 19.2 °C | 17.8 °C | 38.1 | 0.925 |
| **26** | 3917-3939 | UAUUAACAGCAGCUAAACCAU  GGUUUAGCUGCUGUUAAUAGU | 6.9 °C | 20.9 °C | 35.7 | 1.064 |
| **27** | 4381-4403 | UAUAGAACCAGUACAGUAGGU  CUACUGUACUGGUUCUAUACC | 14.5 °C | 20.2 °C | 40.5 | 0.906 |
| **28** | 4400-4422 | UAAGACAAACACUACAAGGUA  CCUUGUAGUGUUUGUCUUAGU | 19.2 °C | 16.4 °C | 35.8 | 0.951 |
| **29** | 4739-4761 | AAUUACAACCGUCUACAACAU  GUUGUAGACGGUUGUAAUUCA | 6.9 °C | 20.3 °C | 35.8 | 0.968 |
| **30** | 4793-4815 | UACAUUCGACUCUUGUUGCUC  GCAACAAGAGUCGAAUGUACA | 21.1 °C | 16.7 °C | 42.9 | 0.934 |
| **31** | 4827-4849 | UAAAAGGACCUUCUAACACCA  GUGUUAGAAGGUCCUUUUAUG | 18.7 °C | 20.3 °C | 38.1 | 0.949 |
| **32** | 4875-4897 | UUCCAAUUGUGUAGUUUGCAA  GCAAACUACACAAUUGGAAUU | 20.1 °C | 11.8 °C | 33.33 | 0.941 |
| **33** | 5057-5079 | UCUUUUGACCAGCUUUAUCAA  GAUAAAGCUGGUCAAAAGACU | 12.2 °C | 13.6 °C | 35.7 | 1.064 |
| **34** | 5085-5107 | AAAUGAGAGAGAGAAUGUCUU  GACAUUCUCUCUCUCAUUUUG | 20.4 °C | 19.2 °C | 35.7 | 0.926 |
| **35** | 5202-5224 | UAAACAGACGCUGAUUUUGCA  CAAAAUCAGCGUCUGUUUACU | 19.2 °C | 7.4 °C | 38.1 | 0.966 |
| **36** | 5230-5252 | UAUAGGUUGACACAUAAGCUG  GCUUAUGUGUCAACCUAUACU | 18.5 °C | 11.6 °C | 38.1 | 1.020 |
| **37** | 5351-5373 | UGAGUUUUUCCAUUGGUACGU  GUACCAAUGGAAAAACUCAAA | 13.3 °C | 20.0 °C | 35.7 | 0.944 |
| **38** | 5407-5429 | AUUGUCUAAGGACACAUUCUU  GAAUGUGUCCUUAGACAAUGU | 20.3 °C | 19.3 °C | 35.7 | 0.933 |
| **39** | 5514-5536 | UCUAUGUCAGAUUGAUGUGAC  CACAUCAAUCUGACAUAGAAG | 20.3 °C | 20.5 °C | 38.1 | 0.975 |
| **40** | 5698-5720 | UAGUUGUUCAGACAAUGACAU  GUCAUUGUCUGAACAACUACG | 17.8 °C | 20.5 °C | 38.1 | 0.985 |
| **41** | 5706-5728 | UGUUUUCGUAGUUGUUCAGAC  CUGAACAACUACGAAAACAAA | 15.3 °C | 20.5 °C | 35.7 | 0.982 |

| **Table S5.** Target position, target sequence, RNA oligo sequences, seed-duplex stability (Tm), GC content, and binary validity scores for predicted siRNA molecules for targeting NSP4 of SARS-CoV-2. | | | | | | |
| --- | --- | --- | --- | --- | --- | --- |
| **No.** | **Target position** | **RNA oligo sequences**  **21nt guide (5′→3′)**  **21nt passenger (5′→3′)** | **Seed-duplex stabilty (Tm)** | | **GC%** | **Binary Validity** |
|  |  |  | **Guide** | **Passenger** |  |  |
|  |  |  | **°C** | **°C** |  |  |
| **1** | 84-106 | UUAGACAUGACAUGAACAGGU  CUGUUCAUGUCAUGUCUAAAC | 20.3 °C | 20.5 °C | 38.1 | 0.855 |
| **2** | 170-192 | AACAAGUAUCUGUAGAUGCUA  GCAUCUACAGAUACUUGUUUU | 19.0 °C | 20.3 °C | 33.33 | 0.923 |
| **3** | 253-275 | AAUCAAUGGGCAAGCUUUGUC  CAAAGCUUGCCCAUUGAUUGC | 13.6 °C | 17.0 °C | 45.24 | 0.846 |
| **4** | 324-346 | UUAGUUGUGCGUAAUAUCGUG  CGAUAUUACGCACAACUAAUG | 19.0 °C | 3.5 °C | 38.1 | 1.065 |
| **5** | 394-416 | UUUUGAUGGUGUGUAACAGAU  CUGUUACACACCAUCAAAACU | 13.8 °C | 20.4 °C | 35.7 | 0.935 |
| **6** | 434-456 | AAACACAAGCUGAUGUUGCAA  GCAACAUCAGCUUGUGUUUUG | 19.3 °C | 20.5 °C | 40.8 | 0.908 |
| **7** | 613-635 | UCUAACAGAACCUUCAAGGUA  CCUUGAAGGUUCUGUUAGAGU | 16.4 °C | 16.6 °C | 40.5 | 0.917 |
| **8** | 692-714 | UAGUAGAUACACAAACACCAG  GGUGUUUGUGUAUCUACUAGU | 18.9 °C | 19.3 °C | 38.1 | 1.001 |
| **9** | 713-735 | UGUUAAGUACCCAUCUACCAC  GGUAGAUGGGUACUUAACAAU | 12.9 °C | 20.3 °C | 40.5 | 1.024 |
| **10** | 832-854 | UAUAGAUGCUGAUAUGUCCAA  GGACAUAUCAGCAUCUAUAGU | 13.3 °C | 16.0 °C | 35.7 | 0.998 |
| **11** | 1419-1441 | UUACUGAAGUCAUUGAGAGCC  CUCUCAAUGACUUCAGUAACU | 20.3 °C | 20.4 °C | 40.5 | 0.939 |
| **12** | 1423-1445 | UGAGUUACUGAAGUCAUUGAG  CAAUGACUUCAGUAACUCAGG | 21.4 °C | 19.2 °C | 40.5 | 0.940 |

| **Table S6.** Target position, target sequence, RNA oligo sequences, seed-duplex stability (Tm), GC content, and binary validity scores for predicted siRNA molecules for targeting NSP6 of SARS-CoV-2. | | | | | | | |
| --- | --- | --- | --- | --- | --- | --- | --- |
| **No.** | **Target position** | **Target sequence**  **21nt target + 2nt overhang** | **RNA oligo sequences**  **21nt guide (5′→3′)**  **21nt passenger (5′→3′)** | **Seed-duplex stabilty (Tm)** | | **GC%** | **Binary Validity** |
|  |  |  |  | **Guide** | **Passenger** |  |  |
|  |  |  |  | **°C** | **°C** |  |  |
| **1** | 292-314 | TTGGATATGGTTGATACTAGTTT | ACUAGUAUCAACCAUAUCCAA GGAUAUGGUUGAUACUAGUUU | 11.2 °C | 21.2 °C | 33.33 | 0.970 |
| **2** | 372-394 | AGCAAGAACTGTGTATGATGATG | UCAUCAUACACAGUUCUUGCU CAAGAACUGUGUAUGAUGAUG | 14.9 °C | 17.7 °C | 38.1 | 0.979 |
| **3** | 783-805 | TAGCATAGATGCCTTCAAACTCA | AGUUUGAAGGCAUCUAUGCUA GCAUAGAUGCCUUCAAACUCA | 14.9 °C | 13.3 °C | 40.48 | 0.919 |

| **Table S7.** ORFs, Target position, target sequence, RNA oligo sequences, seed-duplex stability (Tm), GC content, and binary validity scores for predicted siRNA molecules for targeting NSP6 of SARS-CoV-2. | | | | | | | |
| --- | --- | --- | --- | --- | --- | --- | --- |
| **ORFs** | **Sr. No.** | **RNA oligo sequences 21nt guide (5′→3′) 21nt passenger (5′→3′)** | **Seed-duplex stability (Tm)** | | | **GC %** | **Binary Validity** |
|  |  |  | **Guide** | **Passenger** | |  |  |
|  |  |  | **°C** | | **°C** |  |  |
| **3a** | 1. | UGAAAAACAGCAAGAAGUGCA CACUUCUUGCUGUUUUUCAGA | 8.7 °C | | 17.7 °C | 38.09 | 0.925 |
|  | 2. | AACAACAACAGCAAGUUGCAA GCAACUUGCUGUUGUUGUUUG | 19.3 °C | | 16.7 °C | 40.7 | 0.980 |
|  | 3. | UAUACUCUGCAAGAAGUAGAC CUACUUCUUGCAGAGUAUAAA | 18.9 °C | | 18.9 °C | 35.17 | 0.927 |
|  | 4. | AGAAAUAGGACUUGUUGUGCC CACAACAAGUCCUAUUUCUGA | 6.9 °C | | 19.3 °C | 40.47 | 0.961 |
|  | 5. | UCAGAAAUAGGACUUGUUGUG CAACAAGUCCUAUUUCUGAAC | 12.0 °C | | 17.8 °C | 38.09 | 0.947 |
|  | 6. | AGUAUAACCACCAAUCUGGUA CCAGAUUGGUGGUUAUACUGA | 9.5 °C | | 18.1 °C | 40.47 | 1.020 |
|  | 7. | AAAUUGGUUCCAUUACUGGAU CCAGUAAUGGAACCAAUUUAU | 18.8 °C | | 11.6 °C | 33.33 | 0.992 |
| **6** | 1. | AAUCAAUCUCCAUUGGUUGCU CAACCAAUGGAGAUUGAUUAA | 16.2 °C | | 1. °C | 35.71 | 0.963 |
| **7a** | 1. | UUAUCAGUGCCAAGAAAAGAA CUUUUCUUGGCACUGAUAACA | 20.3 °C | | 5.5 °C | 35.71 | 0.891 |
|  | 2. | UUUUGAGUGUGAAGCAAAGUG CUUUGCUUCACACUCAAAAGA | 19.2 °C | | 18.6 °C | 38.09 | 0.921 |
| **8** | 1. | UACAUUCUUGGUGAAAUGCAG GCAUUUCACCAAGAAUGUAGU | 19.2 °C | | 13.8 °C | 38.09 | 0.955 |
|  | 2. | UUGAUGUUGAGUACAUGACUG GUCAUGUACUCAACAUCAACC | 20.5 °C | | 21.5 °C | 40.47 | 0.949 |
|  | 3. | AUAUCGAUGUACUGAAUGGGU CCAUUCAGUACAUCGAUAUCG | 17.8 °C | | 18.1 °C | 40.47 | 0.890 |
|  | 4. | UUCAUAGAACGAACAACGCAC GCGUUGUUCGUUCUAUGAAGA | 21.4 °C | | 19.8 °C | 42.85 | 0.940 |
| **10** | 1. | AUAUCGUAAACGGAAAAGCGA GCUUUUCCGUUUACGAUAUAU | 17.7 °C | | 18.7°C | 35.71 | 0.924 |

| **Table S8.** Key to nucleic acid geometry cutoffs and goals in MolProbity Multi Criterion Report. | |
| --- | --- |
| **Nucleic acid geometry** | **Goal** |
| Probably wrong sugar puckers | 0 |
| Bad backbone conformations | <=5% |
| Bad bonds | 0% |
| Bad angles | <0.1% |
